# Cation‐Anchoring‐Induced Efficient n‐Type Thermo‐Electric Ionogel with Ultra‐High Thermopower

**DOI:** 10.1002/advs.202414389

**Published:** 2025-03-24

**Authors:** Wenchao Zhen, Chengshuai Lu, Duo Li, Guangfan Meng, Hongqin Wang, Yifei Jiang, Jiang Lou, Wenjia Han

**Affiliations:** ^1^ Key Laboratory of Pulp and Paper Science & Technology of Ministry of Education Qilu University of Technology Shandong Academy of Sciences Jinan 250353 P. R. China; ^2^ State Key Laboratory of Biobased Material and Green Papermaking Qilu University of Technology Shandong Academy of Sciences Jinan 250353 P. R. China; ^3^ Yingkou Shengquan High‐tech Materials Co., Ltd Yingkou 115000 P. R. China

**Keywords:** high Seebeck coefficient, ionic liquids, ionogel thermo‐electric materials, multi‐functional sensors, polyacrylic ester

## Abstract

Ionogels have emerged as promising candidates for low‐grade thermal energy harvesting due to their leak‐free electrolytes, exceptional flexibility, thermal stability, and high thermopower. While substantial progress in the thermoelectric performance of p‐type ionogels, research on n‐type ionic materials lags behind. Striking a harmonious balance between high mechanical performance and thermoelectric properties remains a formidable challenge. This work presents an advanced n‐type ionogel system integrating polyethylene glycol diacrylate (PEGDA), hydroxyethyl methacrylate (HEMA), 1‐allyl‐3‐methylimidazolium chloride ([AMIM]Cl), and bacterial cellulose (BC) through a rational design strategy. The synergistic combination of photo‐polymerization and hydrogen‐bonding networks effectively immobilizes imidazolium cations while enabling rapid chloride ion transport, creating a pronounced cation‐anion mobility disparity that yields a substantial negative ionic Seebeck coefficient of −7.16 mV K⁻¹. Furthermore, BC's abundant hydroxyl groups establish multivalent hydrogen bonds within the ternary polymer matrix, endowing the composite with exceptional mechanical properties—notably a tensile strength of 3.2 MPa and toughness of 4.1 MJ m⁻^3^. Moreover, the ionogel exhibits sensitive responses to stimuli such as pressure, strain, and temperature. The thermoelectric modules fabricated can harness body heat to illuminate a bulb, showcasing great potential for low‐grade energy harvesting and ultra‐sensitive sensing.

## Graphical abstract

An n‐type thermoelectric ion gel with excellent thermoelectric, mechanical, and sensing properties is fabricated via photopolymerization, leveraging an imidazole cation anchoring strategy to enhance thermoelectric performance and multiple hydrogen bonds from bacterial cellulose to improve mechanical strength.

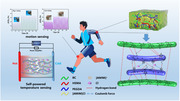

## 1 Introduction

Thermoelectric materials leveraging the Soret effect have garnered significant interest in low‐grade thermal energy conversion and wearable smart sensing applications.^[^ ^1^ ^]^ In contrast to conventional electronic thermoelectric systems, ionic thermoelectric materials have emerged as particularly promising due to their exceptional ionic Seebeck coefficients—reaching magnitudes as high as 10 mV K⁻¹.^[^ ^2^ ^]^ This intrinsic property enables the direct generation of enhanced output voltages and power densities, effectively bypassing the complex multi‐unit integration typically required in electronic counterparts.

Among emerging ionic thermoelectric systems, ionic liquid (IL)‐derived ionogels stand out as particularly compelling candidates, combining quasi‐solid structural integrity with leak‐free operation, mechanical compliance, thermal/chemical stability, and scalable processability.^[^ ^3^ ^]^ The thermodiffusion behavior in these systems is fundamentally governed by the molecular architecture of constituent ions (both cations and anions) and their dynamic interactions with the polymer matrix—particularly through coordination bonds and electrostatic forces. Recent advances in p‐type ionogel optimization have demonstrated remarkable progress via strategic approaches including: i) heteroionic doping to modulate charge carrier concentration,^[^ ^4^ ^]^ ii) solvent engineering approaches leveraging sol‐gel transitions for percolation control,^[^ ^5^ ^]^ iii) anti‐solvent precipitation techniques enhancing ionic segregation,^[^ ^6^ ^]^ iv) hierarchical electrode architectures minimizing interfacial losses,^[^ ^7^ ^]^ and v) precise tuning of matrix‐ion interactions (e.g., hydrogen bonding, Donor‐Acceptor complexes) to amplify cationic thermophoresis.^[^ ^8^ ^]^ These synergistic strategies have collectively propelled p‐type ionic Seebeck coefficients beyond 15 mV K⁻¹ while maintaining mechanical robustness, establishing new benchmarks for flexible thermoelectric materials.

Nevertheless, the practical implementation of thermoelectric devices necessitates complementary high‐performance p‐type and n‐type units to achieve sufficient energy output,^[^ ^9^ ^]^ yet the development of n‐type ionic thermoelectric materials with comparable Seebeck coefficients significantly lags behind their p‐type counterparts.^[^ ^10^ ^]^ The thermodiffusive behavior of n‐type ionogels is intrinsically governed by ion‐matrix interactions, where strategic molecular engineering of ionic coordination environments has shown promise. Zhao et al. demonstrated that lithium‐ion coordination with ether oxygen atoms in poly(ethylene oxide)‐based matrices enables an exceptional negative Seebeck coefficient of −15 mV K⁻¹, leveraging immobilized Li^+^ cations to amplify anion‐dominated thermophoresis.^[^ ^11^ ^]^ Further advancements by Zhou et al. revealed that electrostatic confinement of methyl viologen (Mfm⁺) cations via poly(styrenesulfonate) (PSS) complexation effectively suppresses cationic migration under thermal gradients, achieving a record ionic Seebeck coefficient of −46.97 mV K⁻¹ in free‐standing films.^[^ ^12^ ^]^ Innovative material design paradigms, including heteroionic doping and asymmetric electrode architectures, have also proven effective. For instance, Le et al. reported a polarity reversal in poly(vinylidene fluoride‐co‐hexafluoropropylene) (PVDF‐HFP)/1‐Butyl‐3‐methylimidazolium tetrafluoroborate (BMIM:BF_4_) ionogels through silver triflate (AgOTF) doping, tuning the Seebeck coefficient from +5.52 to −26.4 mV K⁻¹ via selective anion (BF4⁻)‐matrix interaction modulation.^[^ ^13^ ^]^ Similarly, Cheng et al. achieved dual‐polarity thermal powers (20.2 ± 4 mV K⁻¹ for Cu electrodes versus −10.2 ± 0.83 mV K⁻¹ for activated carbon nanotube (a‐CNT) interfaces in PVDF‐HFP/Sodium trifluoromethanesulfonimide (Na:TFSI) systems, where a‐CNTs’ strong Na⁺ adsorption creates interfacial ion concentration asymmetry.^[^ ^9^ ^]^ Despite these breakthroughs, n‐type ionogel research remains nascent, constrained by limited material diversity and performance metrics that fall short of p‐type benchmarks.^[^ ^14^ ^]^ This disparity critically impedes the development of high‐efficiency ionic thermoelectric modules, as mismatched p‐n pairs suffer from interfacial losses and suboptimal power densities.^[^ ^15^ ^]^ Fundamental challenges persist in engineering ionogels with precisely asymmetric cationic/anionic mobility ratios while maintaining mechanical resilience, highlighting the need for novel polymer architectures capable of simultaneously regulating ion transport and stress dissipation.

Beyond thermoelectric efficiency, the mechanical compliance of ionogel‐based thermoelectric materials,^[^ ^16^ ^]^ particularly their elastic recovery,^[^ ^17^ ^]^ low energy dissipation^[^ ^18^ ^]^ and fatigue resistance^[^ ^19^ ^]^—has become a critical design criterion for next‐generation wearable electronics.^[^ ^20^ ^]^ These requirements stem from operational demands where concurrent maintenance of thermoelectric output stability and interfacial adhesion under dynamic biomechanical stresses remains fundamentally challenging. Recent breakthroughs by Sun et al. exemplify this multifunctional design paradigm, where n‐type iono‐thermal films combining graphene oxide (GO) nanosheets with acrylamide/1‐Ethyl‐3‐methylimidazolium cation ([EMIM]^+^)networks achieved dual breakthroughs: () record negative ionic Seebeck coefficient of −76.7 mV K⁻¹ through π‐cation interactions optimizing [EMIM]^+^ confinement, and ii) mechanical robustness metrics surpassing conventional ionic conductors—219.7 kPa tensile strength, 389% fracture strain, 84.1 kPa Young's modulus, and 0.4 MJ m⁻^3^ toughness—attributed to GO's stress redistribution capability and sacrificial hydrogen bonding.^[^ ^21^ ^]^Nevertheless, Current limitations arise from conflicting design requirements: high ionic mobility for thermodiffusion typically compromises mechanical integrity, while densely crosslinked networks enhancing mechanical properties often restrict ion transport. This performance dichotomy highlights the urgent need for innovative polymer architectures that decouple ionic conduction pathways from structural load‐bearing elements through hierarchical nanostructuring or dynamic bond engineering.

We present a rationally designed n‐type ionogel system fabricated through single‐step photopolymerization of polyethylene glycol diacrylate (PEGDA), hydroxyethyl methacrylate (HEMA), 1‐allyl‐3‐methylimidazolium chloride ([AMIM]Cl), and bacterial cellulose (BC). The strategic immobilization of imidazolium cations via π‐π stacking and Coulombic interactions with the acrylate matrix enables selective chloride anion mobilization, achieving an unprecedented cation/anion thermodiffusivity ratio. This engineered ionic asymmetry yields a high negative ionic Seebeck coefficient of −7.16 mV K⁻¹ at 90% relative humidity (RH). The material's dual‐phase architecture—rigid BC nanofibrils reinforcing a dynamic ionic network—ensures simultaneous preservation of thermoelectric output stability and mechanical integrity. This work establishes a universal paradigm for developing mechanically durable ionic thermoelectrics through controlled ion‐matrix decoupling and nanocomposite design.

## 2 Results and Discussion

### 2.1 Preparation and Mechanism of Ionogels

**Figure 1:**
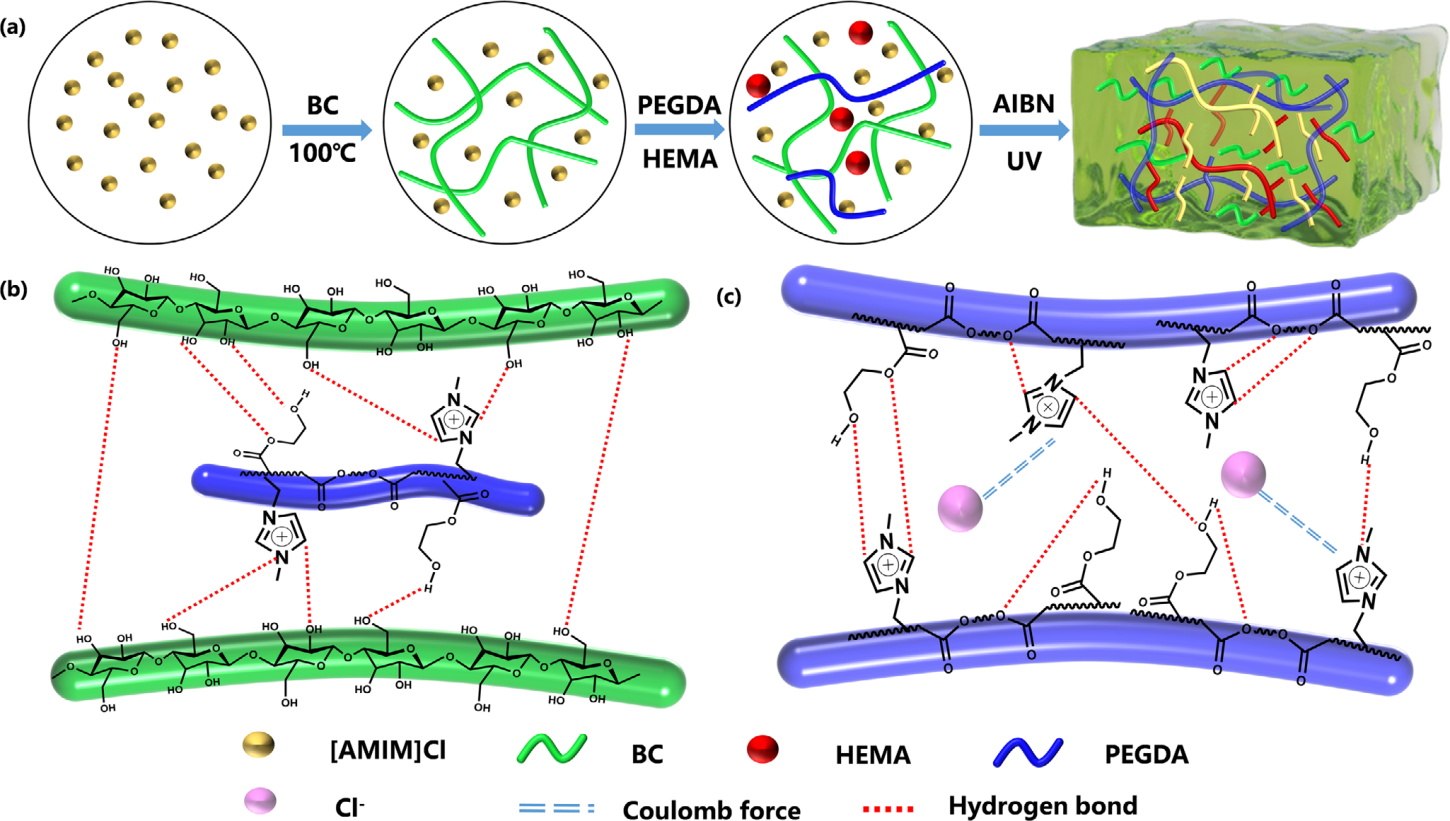
a) Synthesis process of hydroxyethyl methacrylate (HEMA)/polyethylene glycol diacrylic acid (PEGDA)/bacterial cellulose (BC)/ 1‐allyl‐3‐methimidazole chloride ([AMIM]Cl) ionogel. b) Mechanism diagrams of multiple hydrogen bonds in the ionogel between BC and polymer; C)[AMIM] Diagram of the mechanism of hydrogen bonding and Cl‐ migration channels between Cl/PEGDA/HEMA.

The fabrication strategy leverages the exceptional solvation capacity of [AMIM]Cl for BC, enabling complete dissolution via synergistic hydrogen bonding and charge‐transfer interactions.^[^ ^22^ ^]^ Subsequently, PEGDA and HEMA were introduced into the homogeneous BC/[AMIM]Cl solution under vigorous stirring, followed by UV‐triggered free‐radical polymerization initiated by azobisisobutyronitrile (AIBN) (**Figure** **1**a). This process generated a covalently cross‐linked polymeric backbone through copolymerization of [AMIM]Cl, PEGDA, and HEMA, forming the primary network (Figure S3, Supporting information). This covalent framework not only enhanced mechanical robustness (tensile strength: 3.2 MPa) but also established ion‐conductive pathways critical for thermoelectric functionality (Seebeck coefficient: −7.16 mV K⁻¹). Concurrently, a secondary supramolecular network emerged via multivalent hydrogen bonding between abundant functional groups: i) hydrogen bond donors (─OH in BC/HEMA, N─H in imidazolium) and(Figure 1b–ii) acceptors (C═O in PEGDA/HEMA, C─N in [AMIM]Cl) (Figure 1c). These dynamic interactions, enabled energy dissipation during deformation while maintaining structural integrity, resulting in exceptional toughness (4.1 MJ m⁻^3^). The synergistic interplay of covalent cross‐linking and hydrogen bonding created a hierarchical dual‐network architecture. (Figure S4, Supporting information)This design simultaneously addresses the traditional trade‐off between mechanical strength and ionic conductivity in ionogels, achieving a unique combination of high elastic modulus (8.81 MPa) and thermodiffusive ion mobility. This multifunctional ionogel represents a promising platform for flexible electronic skins.

### 2.2 BC Enhances the Mechanical Properties of Ionogels

**Figure 2:**
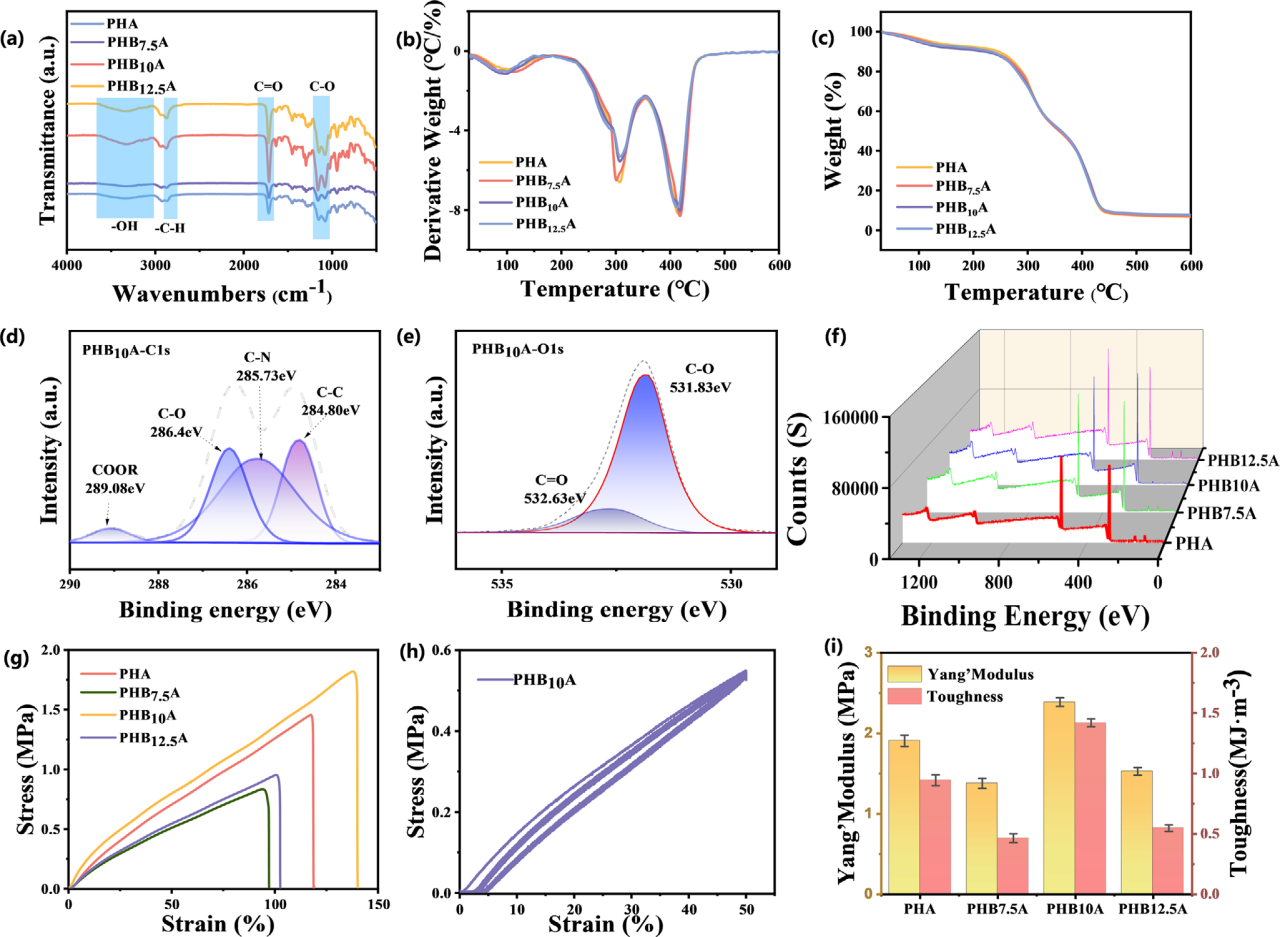
Effects of different BC contents on ionogels. Error bars represent standard deviation; Sample size, *n*  =  3. a) Fourier transform infrared (FTIR) spectroscopy of the PHB_X_A ionogel group. b) Differential Thermal Gravity (DTG) analysis data of samples with different BC additions. c) Thermal Gravity Analysis (TG) analysis data of samples with different BC additions. d) Carbon spectrum from X‐ray photoelectron spectroscopy (XPS) of the PHB_10_A ionogel.e) Oxygen spectrum from X‐ray photoelectron spectroscopy (XPS) of the PHB_10_A ionogel.f) Combined carbon, nitrogen, oxygen, and chlorine spectrum from X‐ray photoelectron spectroscopy (XPS) of the PHB_10_A ionogel. g) Stress‐strain data plot for different BC additive amounts. h) Tensile curves of the PHB_10_A ionogel under 10 cycles of 0–50% strain. i) The PHB_X_A group's Young's modulus and toughness.

Fourier‐transform infrared (FTIR) spectroscopy was employed to investigate the evolution of functional groups and intermolecular interactions within the ionogel system as a function of bacterial cellulose (BC) content (**Figure** **2**a). The pristine polyhydroxyalkanoate (PHA) matrix exhibited a broad hydroxyl (─OH) stretching vibration at 3336 cm⁻¹, characteristic of HEMA's hydroxyl groups. Upon incorporation of BC, this peak underwent a redshift to 3327 cm⁻¹ in PHB_10_A, indicative of hydrogen bond formation between BC's hydroxyl groups and the polymer matrix. Similarly, the C─H stretching vibration at 2924 cm⁻¹ in PHA shifted to 2947 cm⁻¹ (Figure S5 Supporting information), confirming extensive hydrogen bonding between BC and imidazolium cations within the polymeric network.^[^ ^23^ ^]^ The carbonyl (C═O) stretching vibration of ester groups appeared at 1717 cm⁻¹, with corresponding C─O stretching observed at 943 cm⁻¹. Both peaks exhibited systematic shifts upon BC addition, reflecting the formation of hydrogen bonds between BC and HEMA/PEGDA/[AMIM]Cl components (Figure S6, Supporting information). Notably, the intensity enhancement of these characteristic peaks correlated directly with increasing hydrogen bond density. Additional spectral features included: ─CH_2_ stretching vibration at 1278 cm⁻¹ C═N stretching of imidazole rings at 1318 cm⁻¹ Cl bending vibration at 1297 cm⁻¹ Cl stretching vibration at 753 cm⁻¹ (Figure S6, Supporting information)These spectroscopic signatures collectively demonstrate the successful integration of BC into the ionogel matrix through extensive hydrogen bonding interactions, which are critical for enhancing both mechanical and thermoelectric properties. The systematic peak shifts and intensity variations provide direct evidence of molecular‐level interactions governing the material's macroscopic performance.

Thermal stability represents a critical performance metric for ionogels (Figure S7, Supporting information), particularly in applications involving thermal energy harvesting and flexible electronics. Thermogravimetric analysis (TGA) revealed three distinct degradation stages (Figure 2b): Derivative thermogravimetry (DTG) analysis confirmed these degradation mechanisms(Figure 2c), with peak minima at 100, 299, and 417 °C corresponding precisely to the inflection points in the TGA curve. The onset decomposition temperature (T = 235 °C) and maximum degradation rate temperature (T = 385 °C) significantly exceed operational requirements for wearable devices (<100 °C), demonstrating exceptional thermal stability for practical implementation.^[^ ^24^ ^]^

XPS analysis results of the PHB_X_A group samples are shown in Figure 2d,f. The C1s, O1s, and XPS total spectra exhibit the presence of C, N, O, and Cl in the ionogel, corroborated by the energy‐dispersive X‐ray spectrum. The fitting of C1s had four peaks at 284.8, 285.73, 286.4, and 289.08 eV, corresponding to the C─C, C─N/C═N (imidazole group), C─O (carbon in alcohol, ether, or carbonyl) and O─C═O (ester group), respectively.^[^ ^25^ ^]^ These bonds confirmed the structural composition of the ionogel and were in agreement with the results of infrared spectroscopy. The peak fitting of O1s exhibited two peaks at 531.83 and 532.63 eV, corresponding to O─C and O═C bonds, respectively, corroborated by C1s data and infrared data.

The mechanical properties of ionogels with varying BC content were systematically evaluated through stress‐strain analysis, Young's modulus determination, and toughness measurements (Figure 2g,i). The pristine PHA sample exhibited a tensile strength of 1.5 MPa and a fracture strain of 118%. Initial incorporation of 7.5 mg BC (PHB_7.5_A) reduced both tensile strength (1.2 MPa) and fracture strain (95%), attributed to BC‐induced disruption of polymer network crystallinity (Figure S8, Supporting information). Optimal mechanical performance was achieved at 10 mg BC loading (PHB_10_A), with tensile strength increasing to 1.8 MPa and fracture strain to 138%. This enhancement resulted from BC‐mediated hydrogen bond diversification. The dynamic hydrogen bonding network enabled rapid molecular chain recovery, yielding exceptional resilience (energy dissipation <5%) during cyclic loading (0–50% strain, ten cycles). However, excessive BC loading (>10 mg) induced nanofibril aggregation, degrading mechanical properties through stress concentration effects.^[^ ^26^ ^]^ This non‐monotonic relationship between BC content and mechanical performance establishes 10 mg as the optimal loading for balancing tensile strength, fracture strain, and fatigue resistance in wearable applications.

### 2.3 Regulating the Mechanical Properties of Ionogels

**Figure 3:**
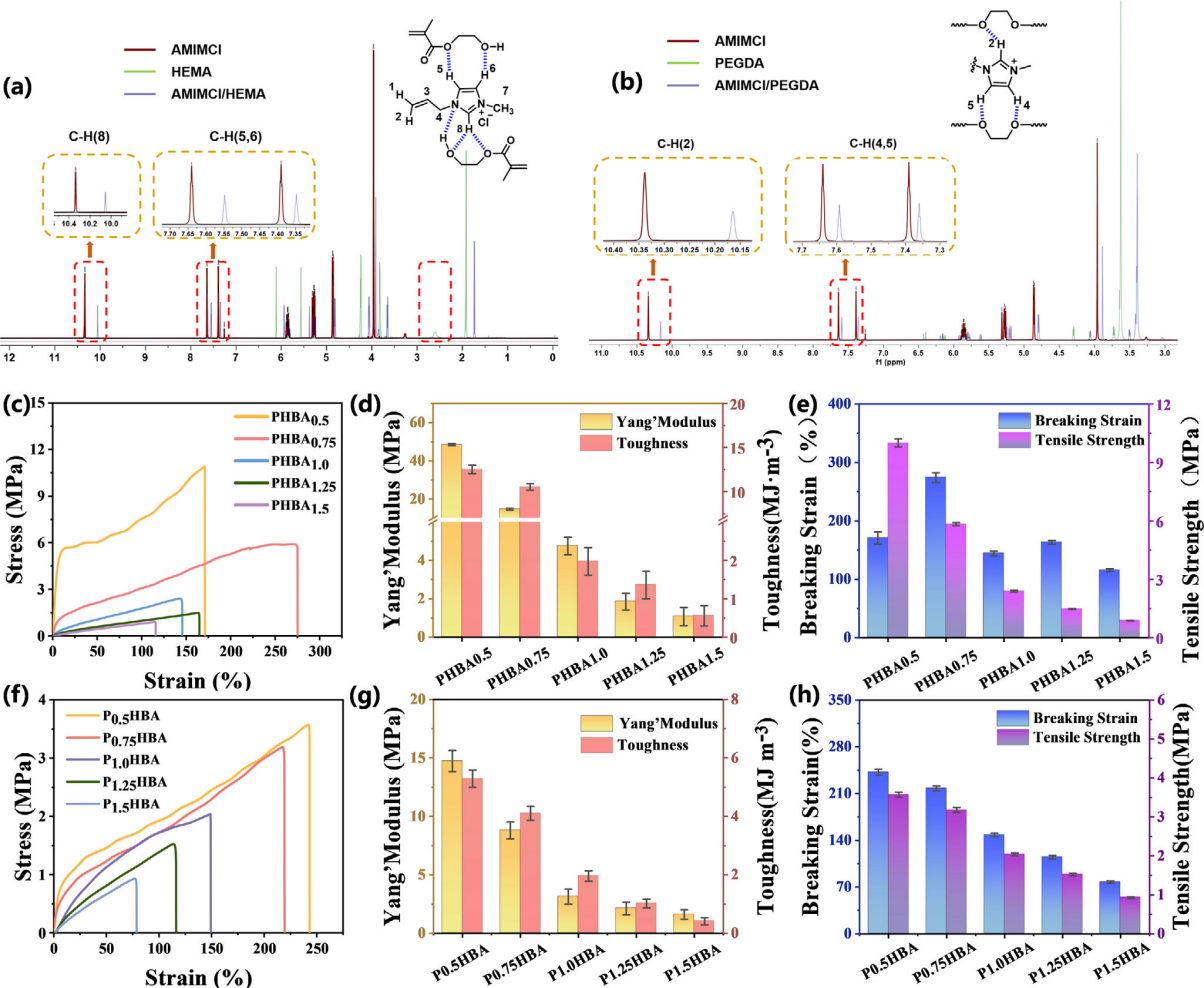
Optimal additions of PEGDA/HEMA and [AMIM]Cl. a) proton nuclear magnetic resonance (H‐NMR) spectra of [AMIM]Cl/HEMA. b) proton nuclear magnetic resonance (H‐NMR) spectra of [AMIM]Cl/PEGDA.c) Stress‐strain curves of the PHBAX ionogel group. d) Young's modulus and toughness data of the PHBAX ionogel group. e) Fracture elongation and tensile strength data of the PHBAX ionogel group. f) Stress‐strain curves of the PXHBA ionogel group. g) Fracture elongation and tensile strength data of the PXHBA ionogel group. h) Young's modulus and toughness data of the PXHBA ionogel group; the total mass of PEGDA and HEMA is 5 g.

To optimize the mechanical properties of ionogels for flexible sensor applications, we systematically modulated the monomer composition to engineer hydrogen bonding networks. The molecular architecture of [AMIM]Cl, PEGDA, and HEMA provides abundant unsaturated bonds and active hydrogen sites, facilitating extensive hydrogen bond formation. Nuclear magnetic resonance (NMR) analysis (**Figure** **3**a,b) revealed key hydrogen bonding interactions:HEMA‐Induced Bonding: Addition of HEMA shifted imidazolium cation resonances (C‐8: 10.06 ppm, C‐5: 7.54 ppm, C‐6: 7.34 ppm),^[^ ^27^ ^]^ indicating hydrogen bond formation between HEMA's hydroxyl groups and imidazolium ring protons. Allyl group electron density in [AMIM]Cl further enabled N1‐HEMA hydrogen bonding. PEGDA‐Induced Bonding: Incorporation of PEGDA caused additional upfield shifts (C‐2: 10.16 ppm, C‐4: 7.58 ppm, C‐5: 7.36 ppm),^[^ ^28^ ^]^ confirming [AMIM]Cl's participation in the polymerization network through multiple hydrogen bonding interactions.Radical copolymerization of HEMA, PEGDA, and [AMIM]Cl enabled precise control over polymer chain length and crosslink density, directly influencing mechanical performance. Systematic variation of [AMIM]Cl content revealed a non‐monotonic relationship between ionic liquid concentration and mechanical properties (Figure 3c,e): Low [AMIM]Cl (0.5 g): Excessive rigidity (Young's modulus = 48.48 MPa) limited strain capacity (<50%), despite high toughness (12.53 MJ m⁻^3^). Optimal [AMIM]Cl (1.25 g) to balanced mechanical performance with high strain capacity (>200%) and minimal energy dissipation (<5%, Figure S9, Supporting information), attributed to optimized hydrogen bond density and distribution.This composition‐dependent mechanical tunability, achieved through controlled hydrogen bonding and crosslinking, establishes 1.25 g [AMIM]Cl as the optimal concentration for flexible sensor applications requiring both mechanical compliance and durability.

The mechanical properties of ionogels were further optimized by systematically varying the PEGDA/HEMA ratio, as shown in Figure 3f,h. Reducing PEGDA content from 1.5 to 0.75 g decreased, corresponding to a reduction in active crosslinking sites. This yielded a more flexible network while maintaining structural integrity. The optimized composition (P_0.75_HBA: 0.75 g PEGDA, 4.25 g HEMA) demonstrated exceptional mechanical performance(Tensile strength: 3.2 MPa Fracture strain: 218%Young's modulus: 8.81 MPa Toughness: 4.1 MJ m⁻^3^). Cyclic loading tests (0–50% strain, 10 cycles) revealed minimal energy dissipation (<10%, Figure S9, Supporting information), indicating excellent elastic recovery. This mechanical optimization stems from balanced crosslinking (PEGDA) and chain flexibility (HEMA), creating a network capable of efficient stress distribution and energy dissipation through reversible hydrogen bonds. The P_0.75_HBA formulation (hereafter denoted as PHBA) was identified as the optimal composition, combining high mechanical resilience with the flexibility required for sensing applications, and excellent thermal stability (Figure S10–S12, Supporting information) Environmental stability (Figures S13, S14, Supporting information) This tailored network architecture enables reliable performance under repeated mechanical deformation, making PHBA particularly suitable for flexible sensing devices requiring both durability and sensitivity.

### 2.4 Thermo‐Electric Properties and Multi‐Functional Applications of PHBA Ionogels

**Figure 4:**
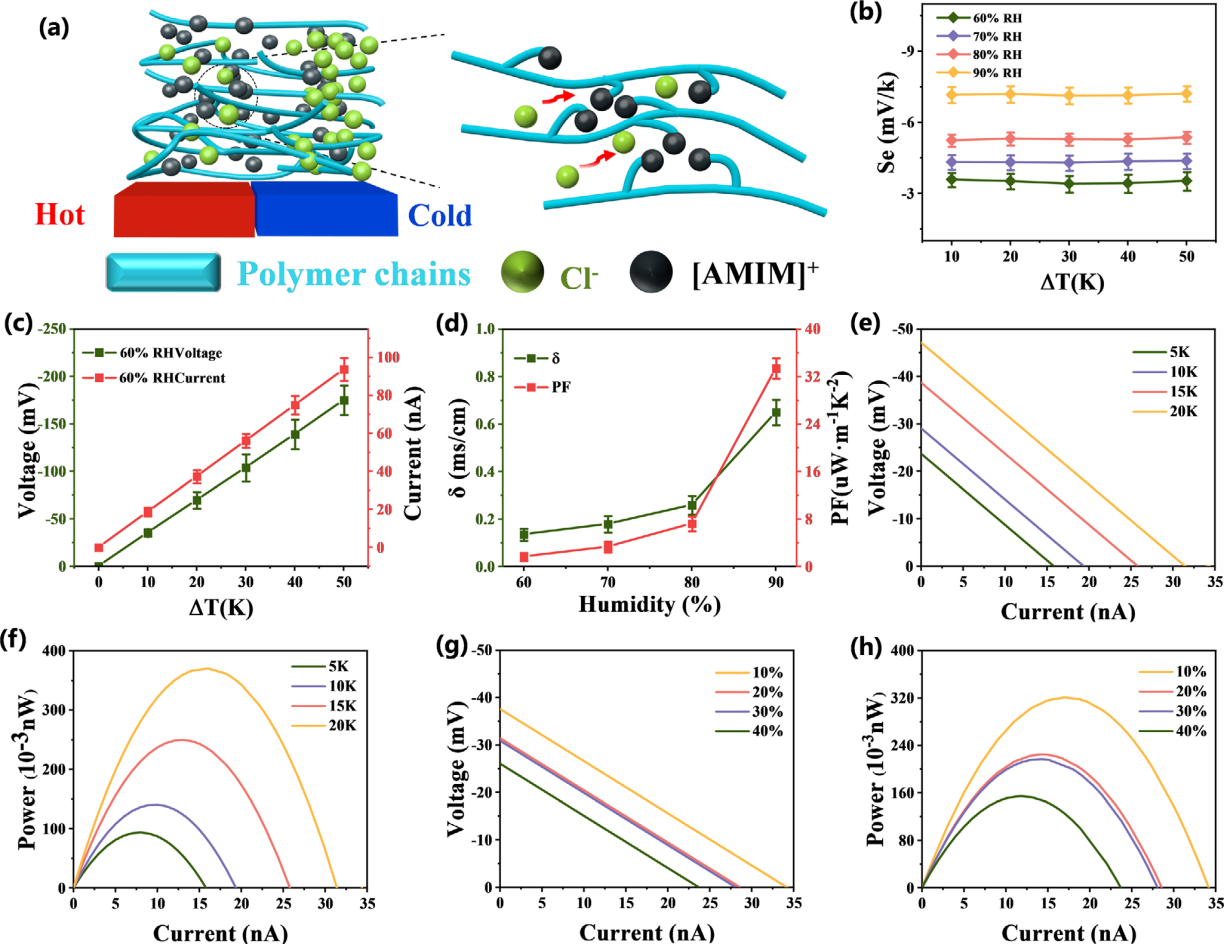
Thermo‐electric performance diagram of PHBA. a) PHBA's thermo‐electric mechanism. b) Seebeck coefficient of PHBA at 60%–90% relative humidity (RH). c) Output voltage/output current of PHBA at 60% RH. d) Conductivity and power factor (PF) of PHBA at different humidities; e) *I–V* curve for PHBA at 60% RH at 5–20 K temperature difference between both ends of PHBA's. f) I–P curve for PHBA at 60% RH at 5–20 K temperature difference between both ends of PHBA's. g) *I–V* curve for different resistances at 10–40% strain (60% RH, ΔT = 20 K). h) *I–P* curve for different resistances at 10%–40% strain (60% RH, ΔT = 20 K).

The thermoelectric performance of the ionogel was systematically characterized under controlled thermal gradients and humidity conditions (**Figure** **4**a–c). Immobilization of imidazolium cations through covalent bonding to the polymer backbone enabled selective chloride ion migration under thermal stimulation. This asymmetric ion transport, driven by synergistic thermal diffusion and Coulombic interactions, generated a significant potential difference across the material. Quantitative analysis revealed a strong temperature dependence of thermoelectric output: Voltage increased from −35.3 ± 3.85 mV (ΔT = 10 K) to −175.2 ± 15.43 mV (ΔT = 50 K); Current increased from 18.8 ± 2.25 nA (ΔT = 10 K) to 93.8 ± 5.95 nA (ΔT = 50 K). The hydrophilic nature of chloride ions further enhanced ion mobility through hydration effects, prompting an investigation of humidity‐dependent performance (Figure 4d). At 90% relative humidity (RH), PHBA achieved optimal thermoelectric properties: Seebeck coefficient: −7.16 ± 0.34 mV K⁻¹, shows excellent N‐type thermoelectric efficiency, Surpassing the majority of thermoelectric materials of the same type. (Table S2, Supporting information). Ionic conductivity: 0.65 ± 0.05 mS cm⁻¹; Power factor: 33.42 ± 1.7 µW m⁻¹ K⁻^2^. Remarkably, PHBA demonstrated exceptional operational stability (Figures S15, S16, Supporting information) and successfully powered commercial capacitors through harvested thermal energy (Figures S17, Supporting information). This combination of high thermoelectric efficiency and environmental stability establishes PHBA as a promising candidate for self‐powered flexible sensing systems, particularly in applications requiring reliable operation under varying humidity conditions.

The power generation characteristics of the PHBA ionogel were systematically investigated under varying thermal gradients and mechanical strain conditions to evaluate its viability for self‐powered wearable electronics. As illustrated in Figure 4e,f, with the cold side stabilized at 25 °C, the output voltage, current, and power exhibited a strong dependence on both external load resistance and applied temperature differential (ΔT). Increasing ΔT from 10 to 20 K enhanced the open‐circuit voltage from −29.05 to −47.15 mV and short‐circuit current from 19.37 to 34.3 nA, achieving a peak power output of 0.37 ± 0.02 nW at ΔT = 20 K. This performance is consistent with the performance characteristics of currently emerging ion thermoelectric materials. Subsequently, mechanical resilience under deformation is critical for wearable electronics applications. As demonstrated in Figure 4g,h, PHBA maintained stable thermoelectric output under moderate strain (<20%), with voltage and current deviations limited to <5%. Remarkably, even under 40% tensile strain, the material retained functional thermoelectric performance (−26.06 mV, 23.7 nA), while simultaneously exhibiting exceptional thermoelectric stability and volumetric integrity across a broad temperature range (30–70 °C, Figures S11, S16, Supporting information). This remarkable achievement originates from its rationally designed dual‐network architecture, which synergistically integrates covalent crosslinks for structural robustness and dynamic hydrogen bonds for efficient energy dissipation. The covalent framework ensures dimensional stability under thermal stress, while the reversible hydrogen‐bonding network enables rapid recovery of ionic conduction pathways post‐deformation. Such a hierarchical design outperforms conventional rigid thermoelectrics (typically failing at >5% strain) and aligns with emerging strategies for stretchable energy harvesters, achieving a unique balance between mechanical compliance (toughness: 4.1 MJ m⁻^3^) and thermoelectric efficiency (PF: 33.42 µW m⁻¹ K⁻^2^).

**Figure 5:**
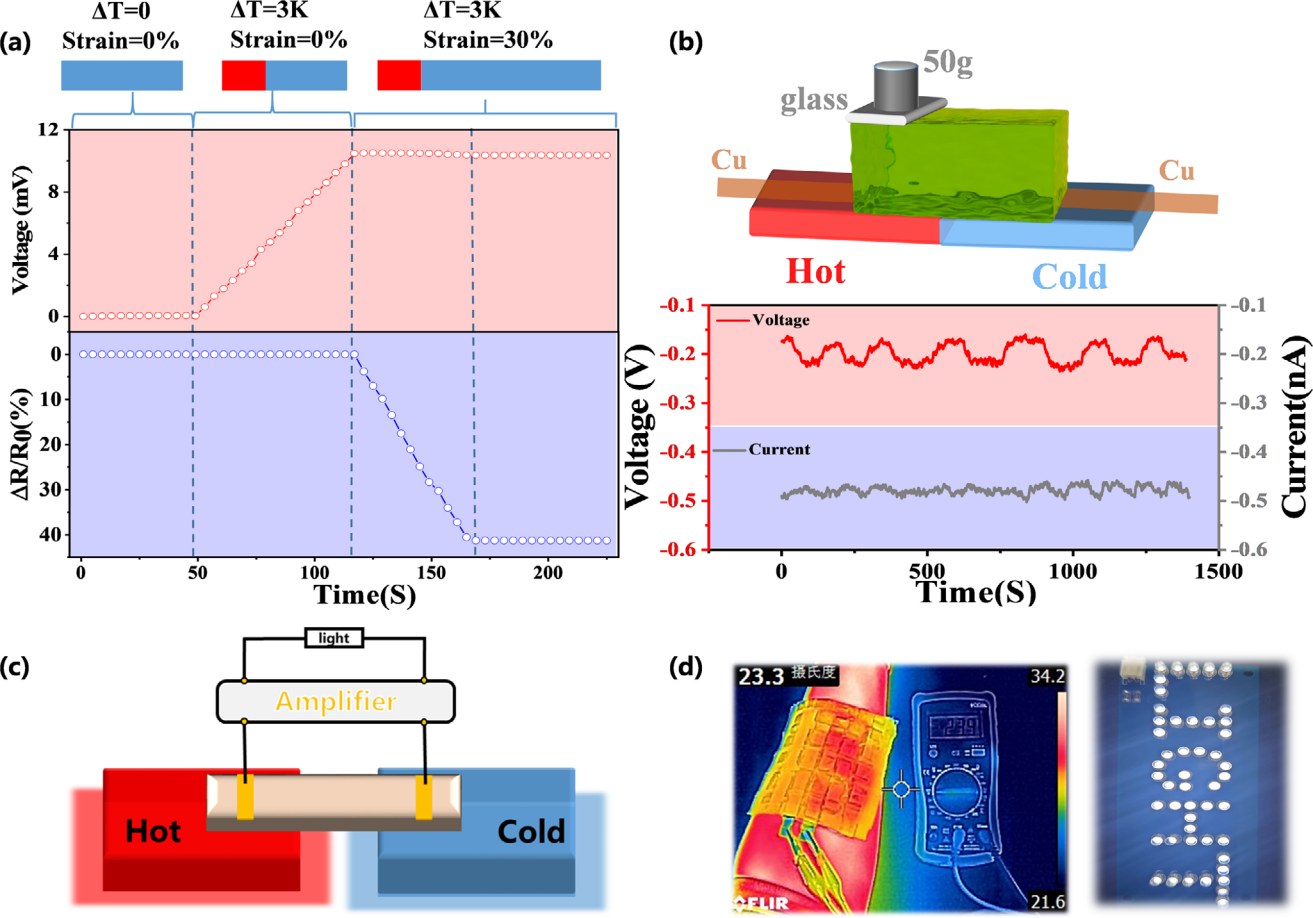
Simulation application of PHBA and the integrated thermo‐electric matrix to achieve thermo‐electric conversion. a) Compound detection of PHBA at 3 K temperature difference and 30% strain. b) PHBA cycle load at 50 g weight at 30 K temperature difference. c) Circuit diagram of PHBA‐integrated device (external power amplifier). d) The PHBA‐integrated ionogel device achieves the illumination of a light bulb by converting and harvesting body heat.

To evaluate the thermoelectric performance under realistic wearable conditions, we simulated various scenarios mimicking human body dynamics (**Figure** **5**a). At 60% relative humidity (RH), a temperature gradient of 3 K and 30% strain were applied to replicate physiological temperature changes during movement.^[^ ^29^ ^]^ During the initial 60 s, the thermal voltage (−10.5 ± 0.3 mV) and relative resistance (ΔR/R₀ = 1.02 ± 0.05) remained stable, demonstrating robust baseline performance. Upon applying the 3 K temperature difference, the thermal voltage gradually increased to −10.5 mV, while the relative resistance exhibited minimal variation, indicating efficient thermoelectric conversion under static conditions. Subsequent application of 30% strain, while maintaining the thermal gradient, revealed a gradual increase in relative resistance (ΔR/R₀ = 1.15 ± 0.06) accompanied by a slight decrease in thermal voltage (−9.8 ± 0.4 mV). Remarkably, the system stabilized within 50 s, maintaining a thermal voltage of −9.5 ± 0.3 mV and a relative resistance of 1.12 ± 0.05, confirming the material's ability to sustain stable self‐powered operation under combined thermal and mechanical stress.

Beyond temperature and strain, pressure stimulation is inevitable in practical applications. As shown in Figure 5b, PHBA's response to pressure was evaluated under a 30 K temperature gradient. Applied pressure enhanced Coulombic interactions between ions, reducing interionic distances and promoting directional chloride ion (Cl⁻) migration along optimized pathways. This resulted in a significant voltage increase and transient current fluctuations, highlighting PHBA's potential for pressure sensing. The synergistic integration of the Seebeck effect and thermal strain response enables the development of multifunctional self‐powered sensing systems capable of simultaneously harvesting energy and detecting environmental stimuli. This dual functionality, combined with PHBA's mechanical resilience and environmental stability, positions it as a versatile platform for next‐generation wearable technologies.

To validate the thermoelectric conversion capability of PHBA in practical applications, we fabricated a power supply matrix comprising 25 individual PHBA units (1 cm × 1 cm × 2 mm) connected in series using highly conductive copper foil to minimize contact resistance (Figure 5c,d). The integrated system, designed to amplify low‐grade thermal energy, employed a power amplifier to boost the output voltage, enabling direct illumination of a commercial LED bulb (Video S1, Supporting information). This demonstration successfully confirmed the efficient conversion of low‐grade heat into usable electrical energy. The scalability of this approach, combined with PHBA's mechanical flexibility and environmental stability, underscores its potential for powering next‐generation wearable electronics. This breakthrough not only validates the material's thermoelectric efficiency but also establishes a practical framework for integrating ionic thermoelectric materials into self‐powered systems, addressing critical challenges in energy autonomy for wearable technologies.

### 2.5 Sensing Properties and Applications of PHBA Ionogel

**Figure 6:**
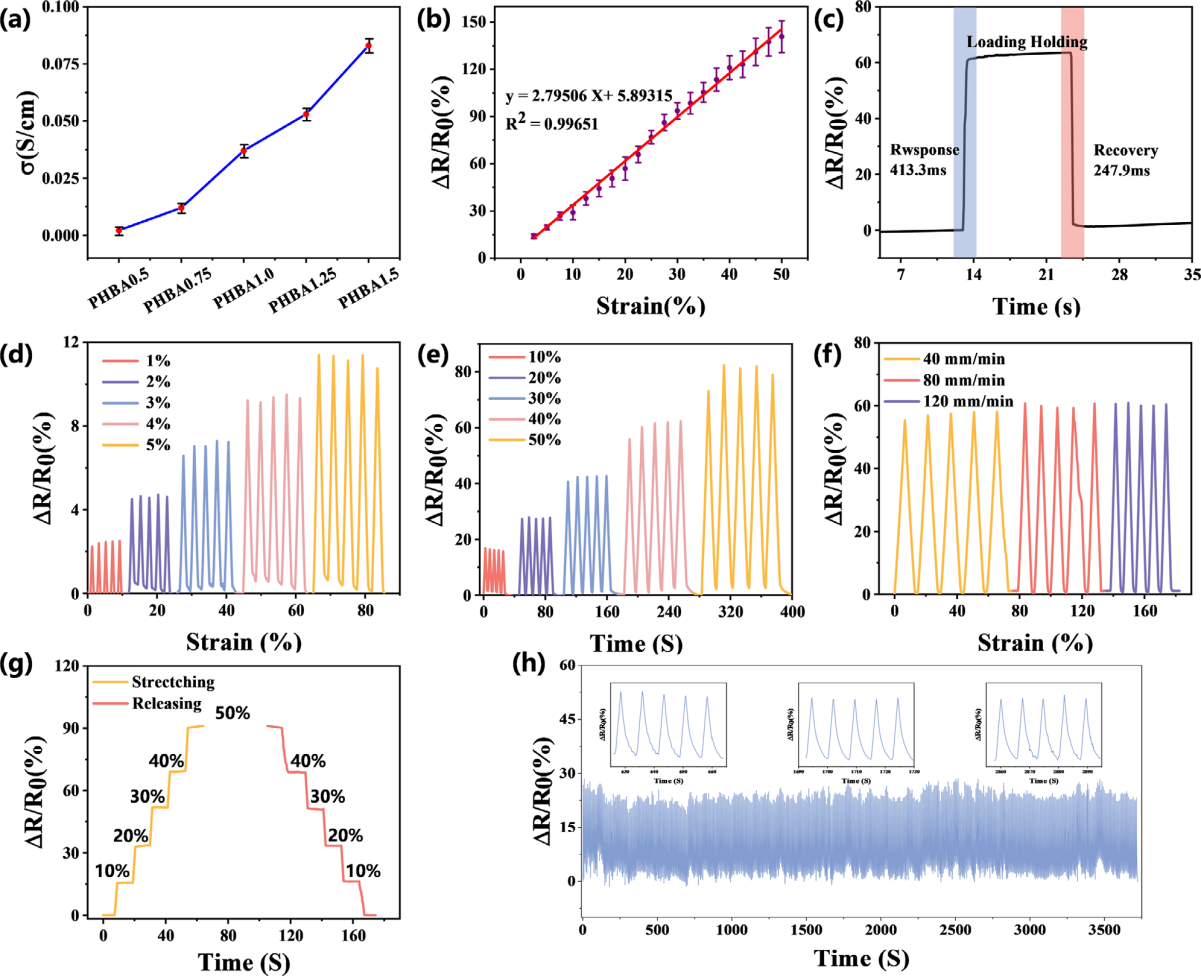
Sensing performance of PHBA. a) Conductivity trend at different [AMIM]Cl ratios. b) Relative resistance change at 0–50% strain. c) Response and recovery times at a single 40% strain. d) Relative resistance change curves of PHBA at 1–5% strain. e) Relative resistance change curves of PHBA at 10–50% strain. f) Different tensile velocity curves of PHBA at 30% strain. g) Instantaneous relative resistance change of PHBA during tensile release. h) Relative resistance change at a 500‐cycle stretch at 10% strain.

To evaluate the sensing capabilities of PHBA ionogels for flexible wearable applications, we systematically characterized three critical performance parameters: ionic conductivity, strain sensitivity, and response dynamics. As shown in **Figure** **6**a, the ionic conductivity exhibited a strong dependence on [AMIM]Cl content, increasing from 0.021 ± 0.018 mS cm⁻¹ to 0.83 ± 0.031 mS cm⁻¹ as the ionic liquid concentration rose from 5 to 30 wt.%. Strain sensitivity, quantified through the gauge factor (GF), demonstrated linear behavior (R^2^ = 0.993) across 0–50% strain, achieving a GF of 2.795 ± 0.039 (Figure 6b)—surpassing conventional carbon‐based strain sensors (GF < 2.5). Temporal resolution tests revealed exceptional response characteristics, with a rapid activation time of 413.3 ms and recovery time of 247.9 ms (Figure 6c), enabling real‐time monitoring of dynamic physiological signals such as joint motion (1–5 Hz) and pulse waveforms. This combination of high conductivity, tunable sensitivity, and ultrafast response kinetics positions PHBA as a versatile sensing platform for next‐generation wearable electronics, addressing the critical need for materials that simultaneously satisfy signal fidelity and mechanical compliance requirements in sports‐monitoring devices.

The strain‐sensing performance of PHBA was further evaluated under diverse mechanical conditions, demonstrating exceptional reliability and durability. As shown in Figure 6d,e, the relative resistance change (ΔR/R₀) exhibited consistent linearity across both low (1–5%) and high strain ranges (10–50%), confirming robust strain sensitivity and operational stability. Notably, the resistance response remained independent of stretching speed (0.1–10 mm s⁻¹), as evidenced by consistent ΔR/R₀ values under varying deformation rates (Figure 6f). The tensile transition profile (Figure 6g) revealed gradual resistance increases with applied strain, highlighting excellent elastic recovery stability.^[^ ^30^ ^]^ Complementary characterization revealed additional functional advantages: strong interfacial adhesion (41.37 ± 1.2 kPa) through hydrogen bonding between ─OH groups and substrates (Figure S18, Supporting information), high optical transparency (>85% at 550 nm) for real‐time visual monitoring (Figure S19, Supporting information). During a continuous cycling experiment of 500 cycles at 10% strain, significant cycling stability was observed (Figure 6h). These properties collectively enable reliable long‐term operation in wearable applications, significantly reducing maintenance requirements while providing consistent signal fidelity for physiological monitoring. The combination of mechanical resilience, optical clarity, and interfacial adhesion positions PHBA as a multifunctional sensing platform capable of addressing the stringent requirements of next‐generation wearable electronics (Table S3, Supporting information).

**Figure 7:**
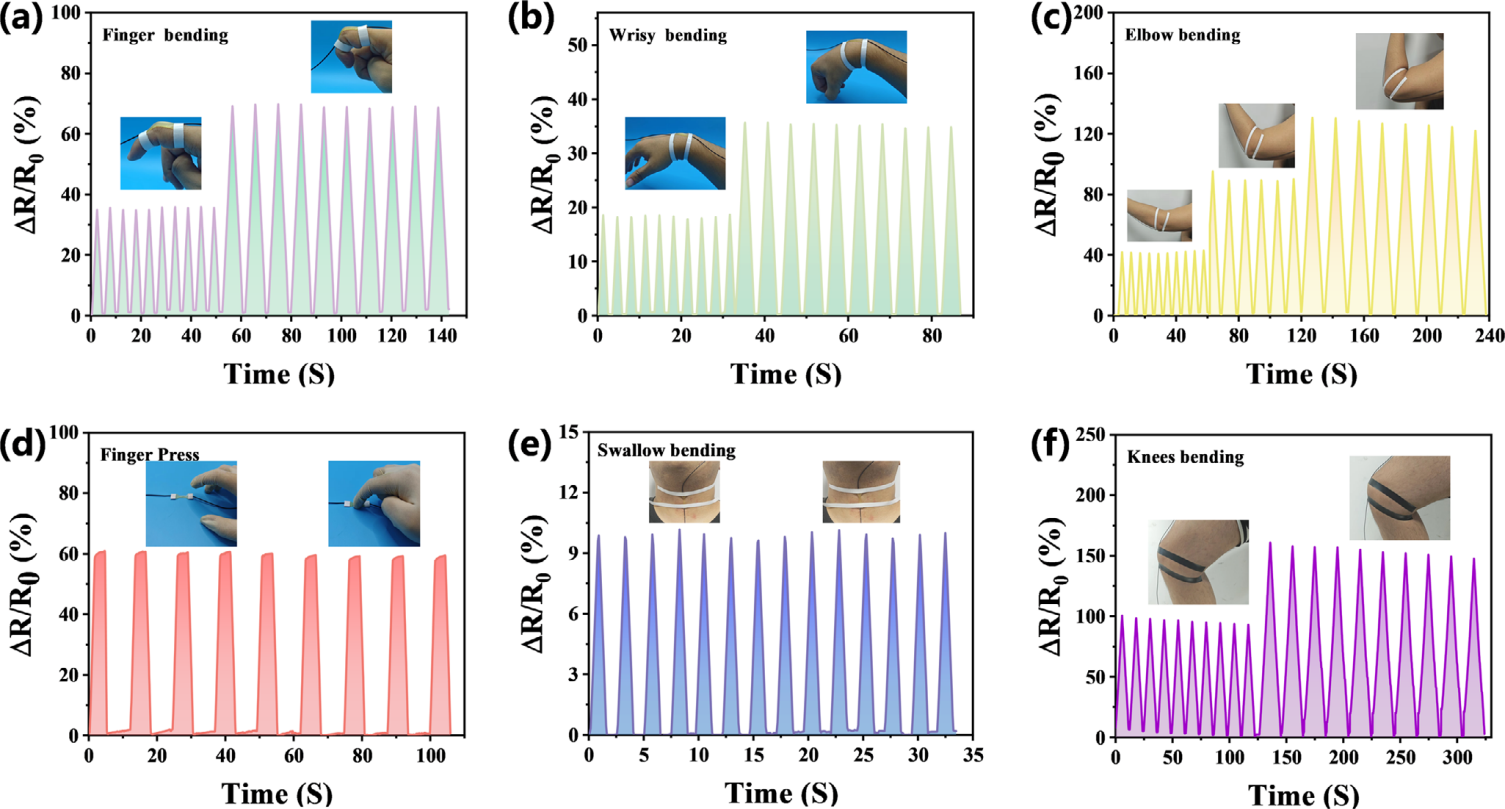
Sensing detection of human movement by the PHBA device. Relative resistance change for a) finger bending, b) wrist bending strain, c) elbow being bent, d) finger pressing, e) during swallowing, and f) standing and sitting down.

The PHBA ionogel sensor, with its good adhesion, excellent mechanical properties, and remarkable sensing performance, has become an ideal wearable sensor for the detection of various physiological activities of the human body. After attaching PHBA to various parts of the human body, it can detect human activities in real‐time and with high accuracy. As can be seen from **Figure** **7**a,d, the relative resistance changes when the finger is bent/pressed, the wrist is bent or the elbow is bent. In terms of motion monitoring, these actions can be accurately and repeatedly tracked in real‐time. This series of phenomena fully proves that this ionogel sensor has great potential application in the field of hand‐motion detection and provides new technical means and possibilities for human motion monitoring and research in related health fields.

Aiming at confirming the ionogel's feasibility for actual detection, we attached it to the throat to detect chewing movements. Figure 7e shows the sensor can transmit similar and accurate signals when accompanied by swallowing movements. This fully indicates that the ionogel sensor can notably detect relatively subtle movements of the human body. As a wearable flexible sensor, it should be able to not only monitor small movements in real time but also accurately detect more intense movements. Figure 7f clearly shows the potential of this sensor in knee‐joint‐motion detection.

This dual capability—capturing micron‐scale throat vibrations (<100 µm displacement) and centimeter‐scale limb motions—surpasses conventional rigid sensors limited to single‐scale detection. The performance stems from PHBA's hierarchical architecture: covalent networks maintain baseline conductivity during large deformations, while dynamic hydrogen bonds amplify nanoscale strain responses. Such multimodal sensing, combined with environmental stability, positions PHBA as a transformative technology for next‐generation wearable systems in healthcare monitoring, sports analytics, and human‐machine interfaces.

## 3 Conclusion

We have developed a novel n‐type ionogel thermoelectric material through rational integration of HEMA, bacterial cellulose (BC), [AMIM]Cl, and PEGDA, achieving breakthrough performance in both energy conversion and mechanical resilience. The strategic immobilization of [AMIM]⁺ cations via hydrogen‐bonded networks and polymer chain entanglement enables selective chloride ion (Cl⁻) mobilization, creating optimized ionic transport channels under Coulombic interactions. This engineered ionic asymmetry, amplified by the Soret effect, yields exceptional thermoelectric performance with a negative Seebeck coefficient of −7.16 mV K⁻¹ and a power factor of 33.42 ± 1.7 µW m⁻¹ K⁻^2^. Simultaneously, the hierarchical dual‐network architecture—comprising covalent crosslinks for structural integrity and dynamic hydrogen bonds for energy dissipation—confers unprecedented mechanical properties: tensile strength of 3.2 MPa, fracture strain of 218%, Young's modulus of 8.81 MPa, and toughness of 4.1 MJ m⁻^3^. The material exhibits ultrafast elastic recovery and cyclic stability, addressing the longstanding trade‐off between flexibility and durability in wearable devices. This work establishes a universal paradigm for designing ionic thermoelectrics that harmonize high energy conversion efficiency, mechanical robustness, and environmental adaptability. The material's multifunctionality opens transformative opportunities in next‐generation wearable healthcare monitors, human‐machine interfaces, and autonomous IoT devices, while its scalable fabrication process aligns with sustainable manufacturing practices.

## 4 Experimental Section

### Materials

BC dispersions were sourced from Qihong Technology Company, Guilin, China. It had a solid content of 1 wt.%, a diameter of 40–60 nm, and an average length of 20 µm. HEMA with 96% purity and containing 250 ppm MEHQ stabilizer, PEGDA(98%) with Mn 600, and 1‐vinyl‐3‐methylimidazolium chloride ([AMIM]Cl)(98%) was purchased from Macklin Chemical Company, Shanghai, China. The photo‐initiator 2,2′‐azobisisobutyronitrile (AIBN) was also obtained from the same source.

### Preparation of Ionogels

First, the BC dispersion was frozen at −20 °C for 30 min and then freeze‐dried at −80 °C for 24 h to obtain freeze‐dried BC. A certain amount of [AMIM]Cl was added to the freeze‐dried BC, followed by placing in a metal bath at 100 °C for 12 h until complete dissolution of BC. After removing from the bath, HEMA, PEGDA, and AIBN were added at room temperature(25 °C), followed by magnetic stirring for 30 min to form a uniformly dispersed solution. A specified amount of this dispersion was placed on a silicone plate and covered with a quartz slice, then exposed to 365 nm, 200 W light‐effect ultraviolet lamp irradiation for 1 min to polymerize it into an ionogel (MC: 3.38 ± 0.23%). Under identical experimental conditions, BC ionogels with different mass fractions and ionogels with varying proportions of HEMA, PEGDA and [AMIM]Cl (Table S1, Supporting information) were prepared for comparison. The letter P stands for PEGDA, H for HEMA, B for BC, and A for [AMIM]Cl. Therefore, the naming convention adopted herein was P_X_HBA/PHB_X_A/PHBA_X_ PHBA, where X represents the amount of the corresponding monomer added when the independent variable is the aforementioned letter. Sample PHBA was selected as the optimal specimen for subsequent testing of thermo‐electric and sensor performances.

### Characterization

The ionogel's chemical structure was characterized using Fourier‐transform infrared spectroscopy (FT‐IR, Nicolet iS5, Thermo Scientific, USA). Nuclear magnetic resonance hydrogen spectroscopy was employed to determine the position and environment of hydrogen atoms in monomer molecules, performed on a Bruker nuclear magnetic resonance instrument (Bruker, Ascend Evo 1.0 GHz NMR, Germany). The surface morphology and elemental map of the freeze‐dried conductive ionogel were analyzed using scanning electron microscopy (GeminiSEM 300, Zeiss, Germany). X‐ray photoelectron spectroscopy (XPS, Thermo Fisher, USA) was employed to quantitatively analyze the elemental composition on the ionogel's surface, with all sample data corrected against the C1s peak of contaminated carbon (binding energy of ≈284.8 eV). The relative resistance change for PHBA ionogel under the influence of tension was measured using a digital source meter (2450, Keithley, USA). When testing the sensing performance of the ionogel, copper foil was used to wrap both ends of the sample to avoid data inaccuracy due to the crocodile clip's penetration into the ionogel. A copper foil‐wrapped ionogel sample was connected to a digital source meter via crocodile clips to examine the ionogel's sensing performance under the influence of tension.

Copper sheets as electrodes and ionogels as electrolytes were connected to a digital source meter by means of crocodile clips to detect their thermo‐electric properties and obtain their output voltage and output current(Figure S1, Supporting information). For the detection of the Seebeck coefficient, one side of the sample was placed on a heating stage and the other on a radiator. Thereafter, the temperature was controlled on one side through a heater, and measured the potential difference (ΔU) between both ends of the sample. The Seebeck coefficient was accordingly calculated.

First, a polyethylene (PE) film was laid in the flat position on a glass plate, stuck a layer of traceless nano double‐sided tape onto the PE film, cut the PHBA sample into a 1‐cm‐side square, and stuck the sample onto the double‐sided tape. It was covered with 2‐cm‐long, 1‐cm‐wide 25 copper foils in an “工”‐shaped manner and connected in a “Z”‐shaped series to ensure complete contact(Figure S2, Supporting information). After covering it with a layer of traceless nano double‐sided tape, it was wrapped and sealed it with tape to ensure that the humidity of the internal environment remained essentially unchanged.

## Conflict of Interest

The authors declare no conflict of interest.

## Author Contributions

W.Z., C.L., D.L., G.M., and H.W. performed conceptualization, methodology, software, and investigation, and wrote the original draft. Y.J., J.L., and W.H. performed conceptualization, methodology, software, investigation, and resources, and wrote the original draft, Supervision.

## Supporting information



Supporting Information

Supplemental Video 1

## Data Availability

The data that support the findings of this study are available from the corresponding author upon reasonable request.
