# Supplementary material for: Cation‐Anchoring‐Induced Efficient n‐Type Thermo‐Electric Ionogel with Ultra‐High Thermopower
Source: Adv Sci (Weinh). 2025 Mar 24;12(19):2414389. doi: 10.1002/advs.202414389 (PMC12097098; doi:10.1002/advs.202414389)
Supplement: Supplementary file 1 — Supporting Information [file ADVS-12-2414389-s002.docx]

**Supporting information**

**Cation anchoring induced efficient N-type thermoelectric ionogel with ultrahigh thermopower**

*Wenchao Zhen^a,b^, Chengshuai Lu^a,b^, Duo Li^a,b^, Guangfan Meng^b^, Hongqin Wang^c^, Yifei Jiang*^a,b^, Jiang Lou*^a,b^, Wenjia Han*^a,b^*

*a.Key Laboratory of Pulp and Paper Science & Technology of Ministry of Education, Qilu University of Technology, Shandong Academy of Sciences, Jinan 250353, P. R. China*

*b.State Key Laboratory of Biobased Material and Green Papermaking, Qilu University of Technology, Shandong Academy of Sciences, Jinan, 250353, P. R. China*

*c.Yingkou Shengquan High-tech Materials Co., Ltd., Yingkou, 115000, P. R. China*

**Table of Contents**

Supplementary figures:

**Table S1.**Composition of the ionogel

**Table S2** Comparison table of the thermoelectric performance

**Table S3** Comparison table of the sensing performance

**Figure S1.** The map of sensing data detection instrument

**Figure S2.** Physical / schematic diagram of the PHBA sample thermoelectric devices

**Figure S3.** Multiscale morphological characterization of PHBA ionogels.

**Figure S4.** The SEM and EDX data detection of the PHB_10_A sample

**Figure S5.** The Raman data plot for PHB_10_A

**Figure S6.** Infrared Spectroscopy Verification of Hydrogen Bonds in PHBA Ionogels.

**Figure S7.** Effect of BC added on thermal stability of ionogel.

**Figure S8.** Effect of ionogels with different BC additions on crystallinity.

**Figure S9.** Ten cycles of PHBA_X_ and P_X_HBAsamples

**Figure S10.** The DSC (Differential Scanning Calorimetry) and TG (Thermogravimetric Analysis) test data graphs for PHBA samples.

**Figure S11.** Volume stability of PHBA at different temperatures

**Figure S12.** DMA Strain-strain performance of PHBA at different temperatures

**Figure S13.** Solvent resistance of PHBA ionogels

**Figure S14.** Mechanical stability of the PHBA ionogels.

**Figure S15.** Thermal stability of the PHBA ionogels at room temperature

**Figure S16.** Stability of the Seebeck coefficient of the PHBA samples.（a）Stability of the Seebeck coefficient and conductivity over 7 days / 7 hours

**Figure S17.** PHBA charges commercial capacitors after absorbing heat

**Figure S18.** For PHBA adherence testing.

**Figure S19.** The 200 - 800 nm UV-light transmittance of the PHBA.

**Figure S20.** The PHBA load lifts a 1 kg weight.

**Supporting Tables and Figures**

**Composition of the ionogel .**

**Table S1.** Composition of the ionogel

| Name (Abbreviation) | HEMA  (g) | PEGDA  (g) | BC  (mg) | [AMIM]Cl  (g) | AIBN  (g) |
| --- | --- | --- | --- | --- | --- |
| PHA | 4.0 | 1.0 | 0 | 1 | 0.06 |
| PHB_7.5_A | 4.0 | 1.0 | 7.5 | 1 | 0.06 |
| PHB_10_A | 4.0 | 1.0 | 10.0 | 1 | 0.06 |
| PHB_12.5_A | 4.0 | 1.0 | 12.5 | 1 | 0.06 |
| PHBA_0.5_ | 4.0 | 1.0 | 10 | 0.5 | 0.06 |
| PHBA_0.75_ | 4.0 | 1.0 | 10 | 0.75 | 0.06 |
| PHBA_1.0_ | 4.0 | 1.0 | 10 | 1.0 | 0.06 |
| PHBA_1.25_ | 4.0 | 1.0 | 10 | 1.25 | 0.06 |
| PHBA_1.5_ | 4.0 | 1.0 | 10 | 1.5 | 0.06 |
| P_0.5_HBA | 4.5 | 0.5 | 10 | 1.25 | 0.06 |
| P_0.75_HBA | 4.25 | 0.75 | 10 | 1.25 | 0.06 |
| P_1.0_HBA | 4 | 1.0 | 10 | 1.25 | 0.06 |
| P_1.25_HBA | 3.75 | 1.25 | 10 | 1.25 | 0.06 |
| P_1.5_HBA | 3.5 | 1.5 | 10 | 1.25 | 0.06 |
| PHBA | 4.25 | 0.75 | 10 | 0.75 | 0.06 |

**Table S2** Comparison table of the thermoelectric performance

| Ionic Conductors | Seebeck coefficient  (mV K^-1^) | output voltage  （mv） | Power factor  (μW m^-1^ K^-2^) | Reference |
| --- | --- | --- | --- | --- |
| PHBA | -7.16 | 23.9 | 33.42 | This work |
| PVDF-HFP-IL | -4.44 | / | / | [11] |
| PVDF-co-CTFE-IL | 1.06 | 19.5 | / | [12] |
| Janus | 19.5 | 16.5 | / | [13] |
| PEO-Li^+^-IL | -15 | 80 | 37.50 | [14] |
| AA- HFBA- MBA-IL | 28.43 | 27.3 | 2.85 | [15] |
| CBCIG | 11.55 | 40 | 29 | [16] |
| PVDF-HFP- EMIMTFSI | -15~19 | 150 | / | [17] |
| ITESC | 0.55 | 7 | 0.066 | [18] |
| Na: DCA -PVDF-HFP-EMIM:DCA | 45 | / | 18.3 | [19] |
| MA-MMA-IL | -6.3 | 25.1 | 0.0106 | [20] |

**Table S3** Comparison table of the sensing performance

| Ionic Conductors | Stress（MPa） | Stress  （%） | Toughness（MJm^-3^） | Response time  （ms） | Recovery time  （ms） | GF | Cycle stability  （Times） | Reference |
| --- | --- | --- | --- | --- | --- | --- | --- | --- |
| PHBA | 3.2 | 218 | 4.1 | 413.3 | 249.7 | 2.8 | 500 | This work |
| PU-IL | 0.82 | 390 | / | 110 | / | 2.14 | 500 | [1] |
| PTA-PU-IL | 2 | 1000 | 3.1 | 830 | 560 | 2.3 | 9000 | [2] |
| WCA | 0.12 | 540 | / | 37 | / | 1.3 | 5000 | [3] |
| WIPS | 3.41 | 380 | / | 149 | 165 | / | 6000 | [4] |
| P（DEEA-co-IBA）/IL | 0.107 | 250 |  | 100 | 190 | 2.4 | 5000 | [5] |
| BCPs-ILs | 0.34 | 720 | 2.48 | 348 | 359 | 1.85 | 5000 | [6] |
| NFIG | 2.3 | 145 |  | 120 | 80 | / | 5000 | [7] |
| BHIG | 0.17 | 578 | 0.92 | / | / | 3.52 | 100 | [8] |
| QMBF | 1.4 | 1030 | 6.8 | 220 | 170 | 1.0 | 500 | [9] |
| ND-1-1 | 1.75 | 579 | / | / | / | 2.4 | / | [10] |


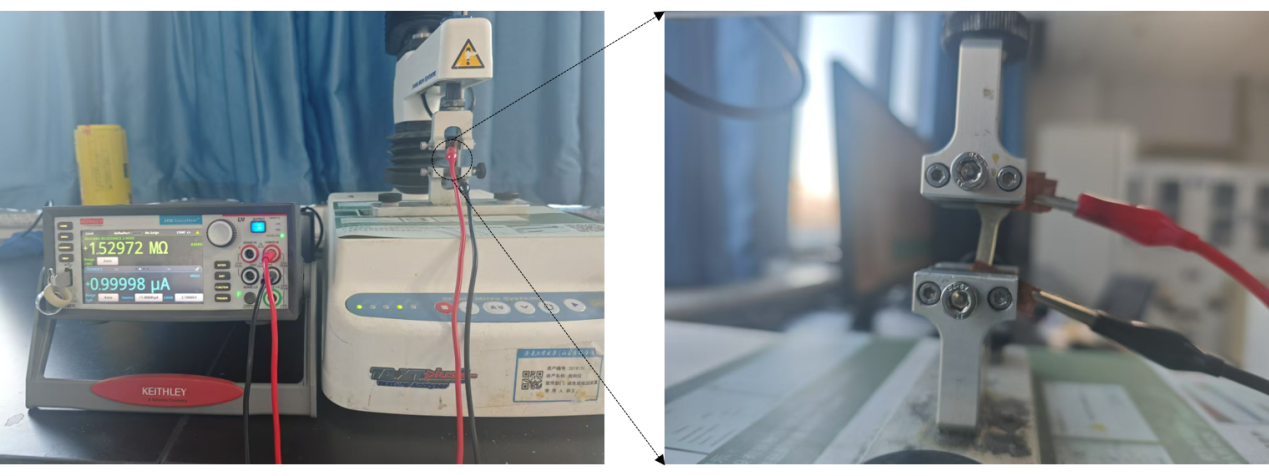


**Figure S1.** The map of sensing data detection instrument

The copper foil-encapsulated PHBA sample was securely clamped in a texture analyzer, and its piezoresistive sensing characteristics were quantitatively evaluated by systematically measuring relative resistance changes under controlled strain levels (1%-50%) and stretching rates (40-120 mm/min). A stepwise loading protocol was implemented to ensure precise strain control.


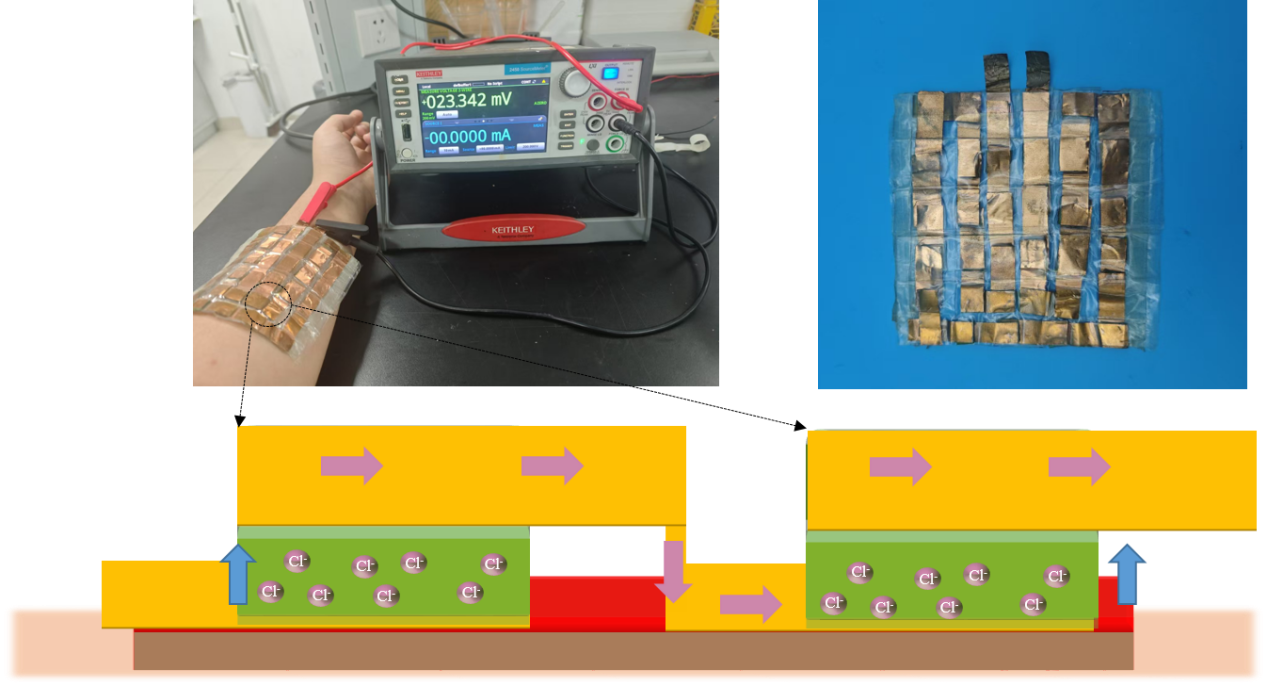


**Figure S2.** Physical / schematic diagram of the PHBA sample thermoelectric devices

As shown in Figure S8, after the PHBA ionogel was configured in series according to previously described steps, it was adhered to human skin. The temperature difference generated by body heat triggered the diffusion of Cl- within the gel to the upper layer under the Seebeck effect, thereby creating a potential difference between the upper and lower layers. Through this series configuration, the internal potential difference of the gel could be maximized.


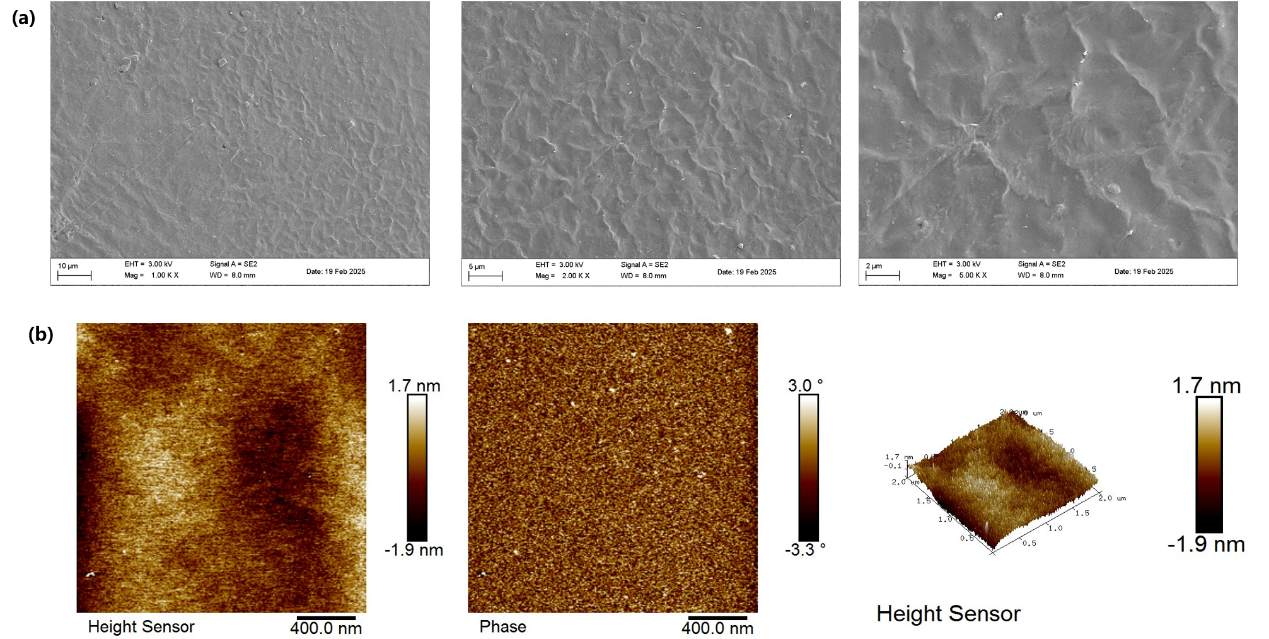


Figure S3. Multiscale morphological characterization of PHBA ionogels. a) Field-emission scanning electron microscopy (FE-SEM) images. b) Atomic force microscopy (AFM) 3D topography .

The morphology of PHBA field-emission scanning electron microscopy at varied magnifications (Figure S3a). High-resolution imaging further showed that the gel surface has a small number of line raised structures, which with the subsequent surface roughness detected by AFM. The atomic force microscopy (AFM) analysis revealed detailed surface characteristics of the sample, as summarized in Figure S3b. The surface exhibited a root mean square roughness (Rq) of 0.516 nm and an average roughness (Ra) of 0.411 nm, indicating a relatively smooth surface morphology. The maximum height difference (Rmax) was measured at 6.47 nm, with a peak height (Rp) of 3.16 nm and a maximum depth (Rv) of -2.32 nm, suggesting the presence of nanoscale surface features. The surface area was calculated to be 4.01 μm², with a minimal surface area difference of 0.179% compared to the projected surface area, highlighting the surface's near-flat topography. The skewness value of -0.145 and kurtosis of 3.21 further describe the asymmetry and distribution of surface heights, respectively. These quantitative measurements provide critical insights into the nanoscale surface properties, which are essential for understanding the material's performance in various applications.


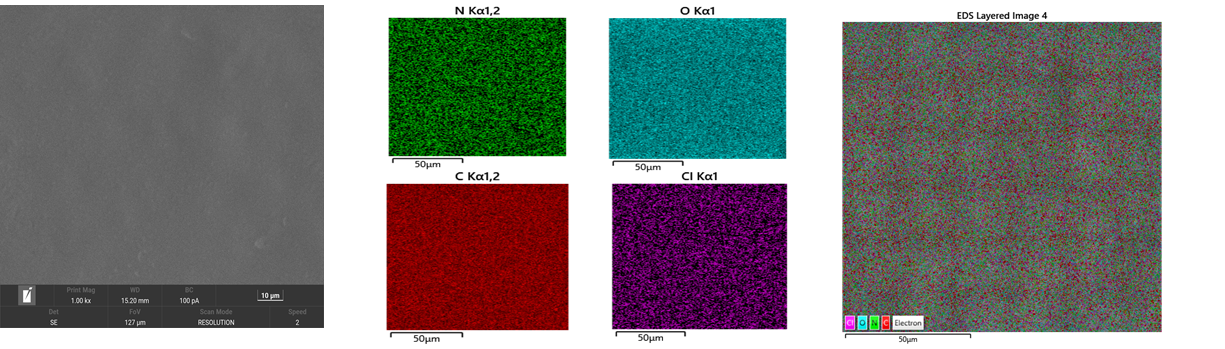


**Figure S4.** The SEM and EDX data detection of the PHB_10_A sample

As shown in Figure S4,Sample PHB_10_A exhibited a smooth surface at 1000 x magnification and according from EDX data indicated that PEGDA / HEMA / [AMIM]Cl / BC was mixed evenly and successfully polymerized in the presence of AIBN, and Check with the infrared spectrum data.


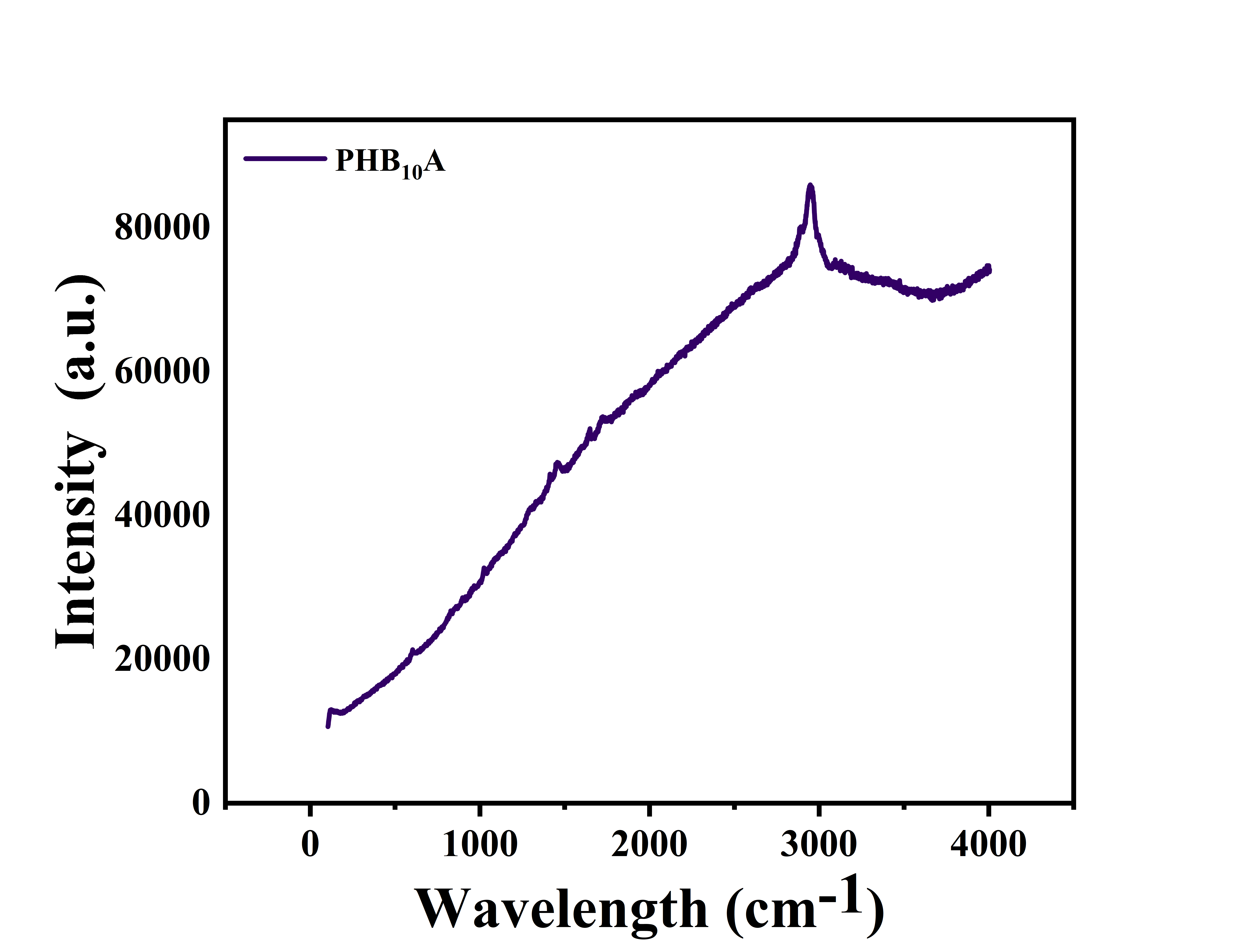


**Figure S5.** The Raman data plot for PHB_10_A

As shown in Figure S5,The absorption peak of the sample PHB_10_A exhibited at 2947cm^-1^ is the C-H absorption peak.


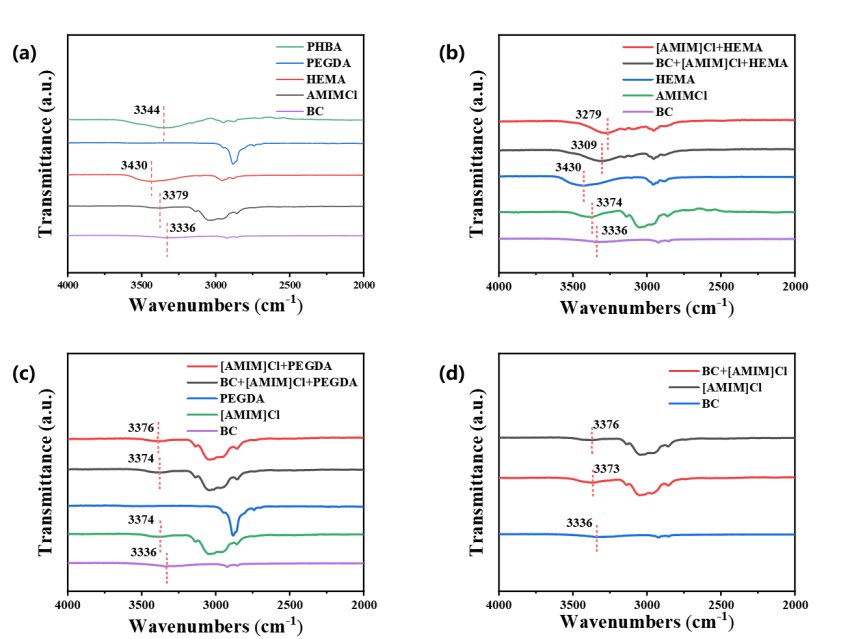


**Figure S6.** Infrared Spectroscopy Verification of Hydrogen Bonds in PHBA Ionogels. (a) FTIR infrared detection of HEMA / PEGDA / AMIMCl / PHBA / BC ; (b-d)Qualitative Infrared Spectroscopic Evidence of Hydrogen Bonding Interaction Between BC and HEMA/PEGDA/[AMIM]Cl

As depicted in Figure S6 (a), we separately detected the infrared transmission spectra of the polymer monomers and the PHBA ionogel. It can be observed from the figure that the -OH stretching vibration peak of PHBA is located at 3344 cm⁻¹. Due to the difficulty in forming uniform mixed solutions of HEMA, PEGDA and BC, we dissolved BC with [AMIM]Cl and then mixed it with HEMA and PEGDA respectively to detect the stretching vibration peaks of -OH therein, aiming to qualitatively express the role of hydrogen bonds among them. As shown in Figure S6 (b - d), both HEMA and PEGDA, along with [AMIM]Cl, have formed hydrogen bonds with BC, which has consequently led to the shift of the -OH absorption peak.


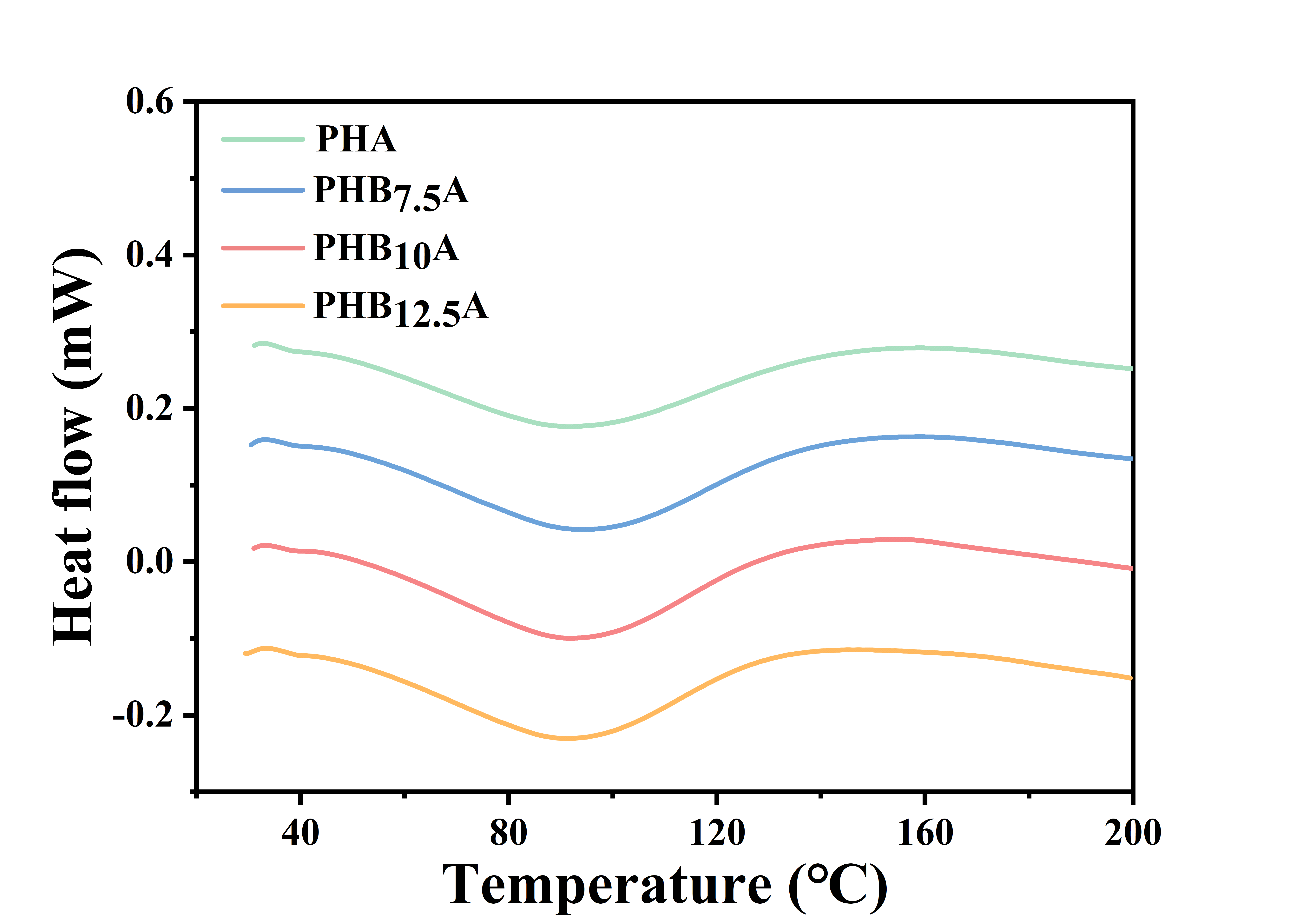


**Figure S7.** Effect of BC added on thermal stability of ionogel.

As shown in Figure S7 Testing of the effect of BC addition on the thermal stability of the sample, ranging from 25℃ to 200 ℃ at 10℃ / min, revealed no significant glass transition temperature. The change of heat flow occurring at 100℃ is the volatilization of moisture.


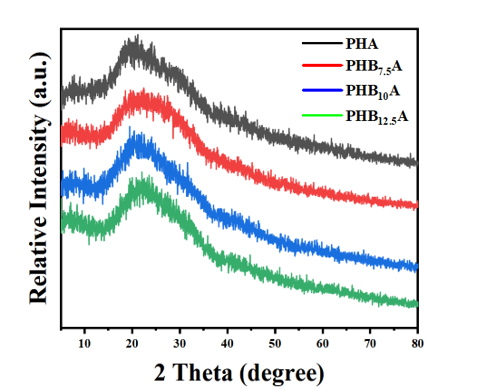


**Figure S8.** Effect of ionogels with different BC additions on crystallinity.

As shown in Figure S8,The crystallinity of PHA was 25.3% without the addition of BC, and the crystallinity of the reaction polymer undergoing crosslinking with HEMA, PEGDA and [AMIM]Cl decreased accordingly, and the hydrogen bonds formed significantly enhanced its mechanical properties. And reached a minimum crystallinity of 3.97% upon addition of 10 mg BC. With the excessive addition of BC, the inhomogeneity of the internal structure of the material increases, thus reducing the overall stress-strain performance of the material.


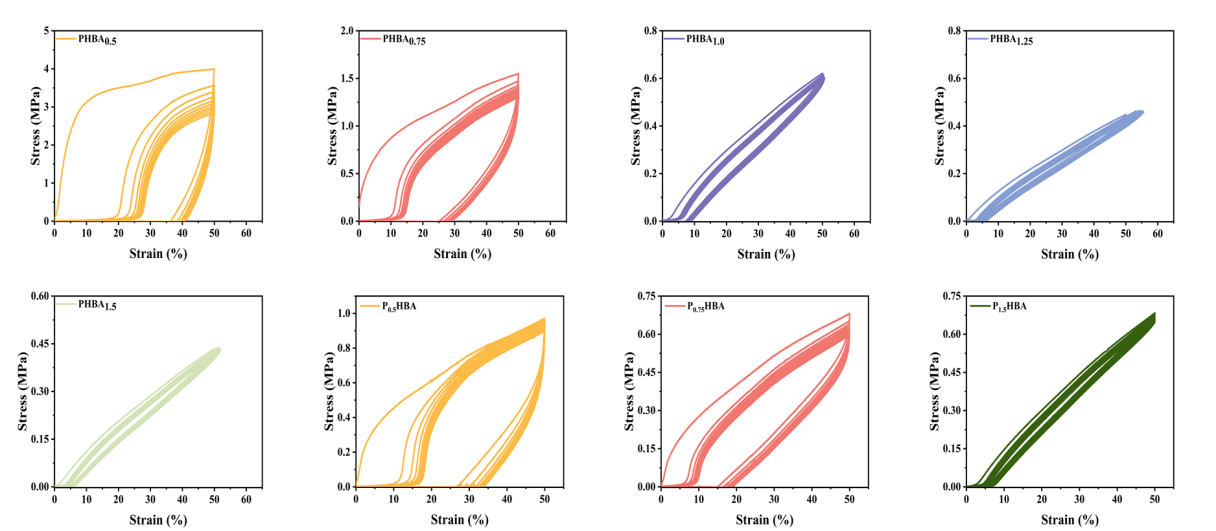


**Figure S9.** Ten cycles of PHBA_X_ and P_X_HBAsamples

As shown in Figure S9, are the ten cycle plots of samples with different AMIMCl and PEGDA / HEMA added. The PHBA_1.25_ cycle lag is small, and the tensile strain is large to meet the needs of human sensing. Therefore, this addition amount was selected for the subsequent experiments. The stress strain was significantly higher in P_0.75_HBA and larger than in P_1.0_HBA and less delayed from the second circle. Therefore, the addition amount ratio was selected as the final sample, namely PHBA.


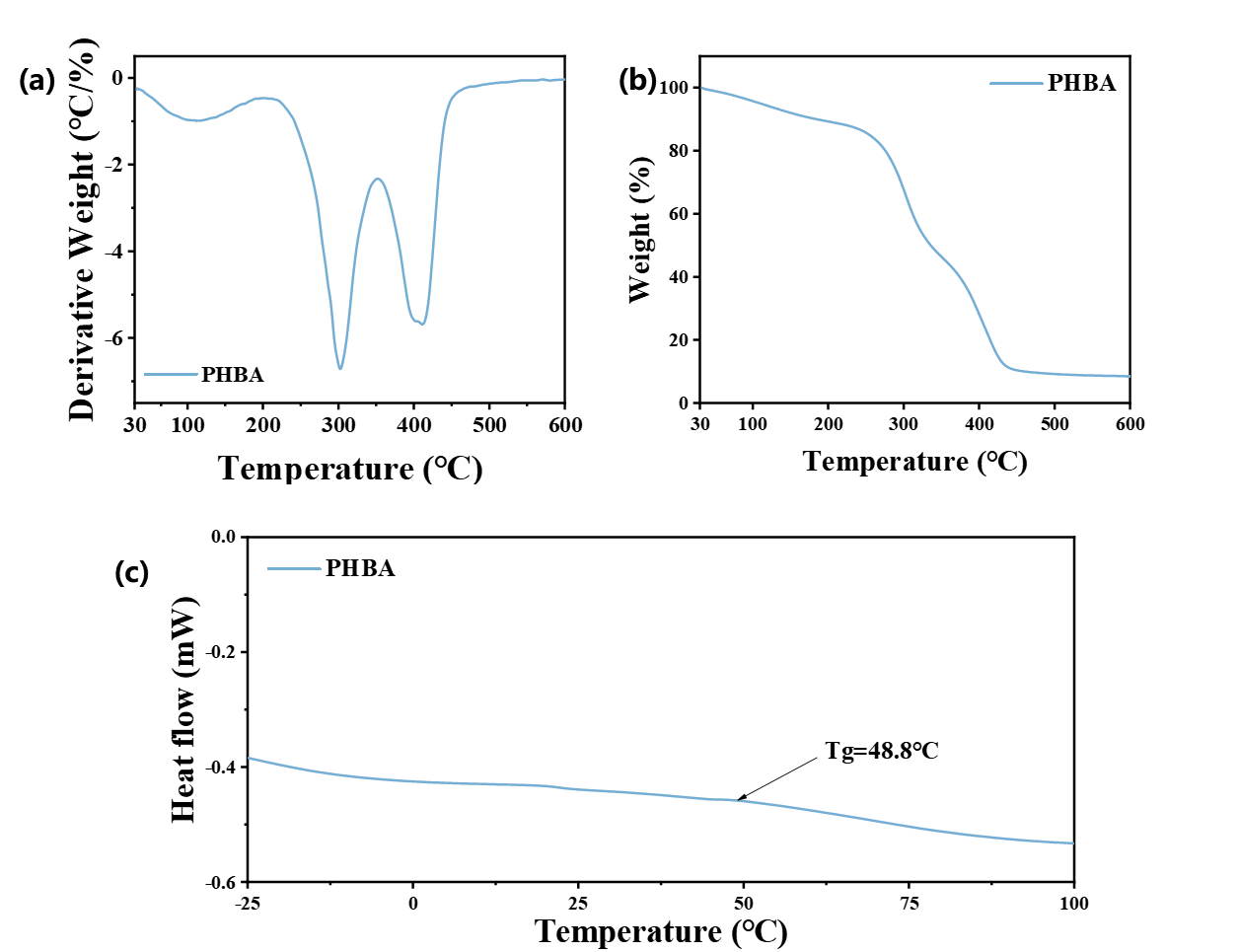


**Figure S10.** The DSC (Differential Scanning Calorimetry) and TG (Thermogravimetric Analysis) test data graphs for PHBA samples

As shown in Figure S10 for the thermal stability of PHBA, the evaporation of water at 100℃ causes the first weight loss, and the other two losses mainly occur between 240-350℃ and 320-420℃. In 20℃, 240-320℃ is mainly caused by the loss of BC in the ionogel; the loss of 320-420℃ is mainly related to the lysis of the polymer backbone and the segmentation of the polymer. The glass transition temperature (Tg) of the PHBA sample has an onset temperature of 48.8°C, a midpoint temperature of 73.07°C, an inflection point at 71.50°C, and an end temperature of 90.82°C. The specific heat change during this transition is 0.333 J/(g·K).


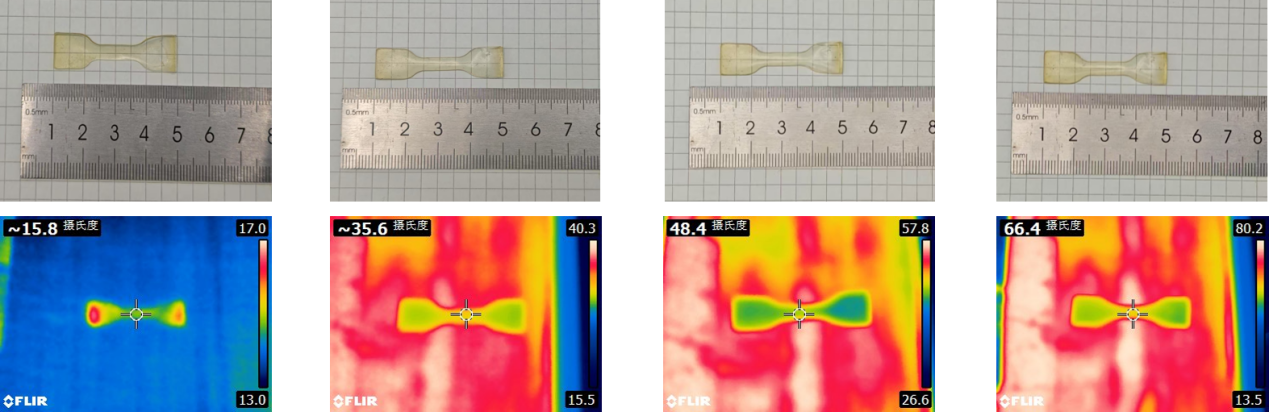


**Figure S11.** Volume stability of PHBA at different temperatures

Precise cutting yielded PHBA ionogel samples of uniform size, which were then heated on heating stages for 30 minutes at room temperature 30°C, 50°C, and 70°C, respectively. Infrared thermal imaging and dimensional change analysis were conducted after the heating. The image data revealed that the volume of the PHBA samples remained essentially unchanged throughout the experimental period, demonstrating excellent volume stability under different temperature conditions.


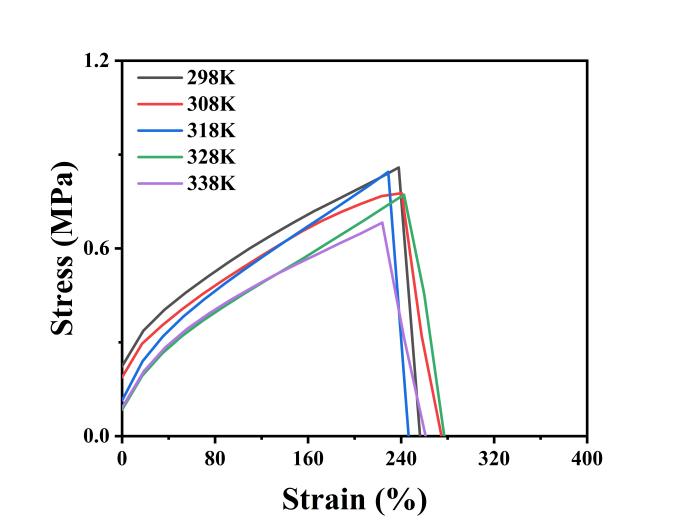


**Figure S12.** DMA Strain-strain performance of PHBA at different temperatures

During the dynamic mechanical analysis (DMA) experiment, the sample undergoes stretching and twisting in the initial stage, resulting in an initial stress that is not zero. Furthermore, because the DMA test employs a dynamic tensile testing method and hydrogen bonding forces diminish under high-temperature conditions, the measured stress values are lower compared to those obtained from mass structural analyzers. Additionally, based on the data derived from dynamic thermodynamic analysis, we observed that PHBA ionogels exhibit high stress-strain properties under various temperature conditions. This discovery reveals the potential for sensing and detection across different temperature environments, thereby further expanding their range of applications.


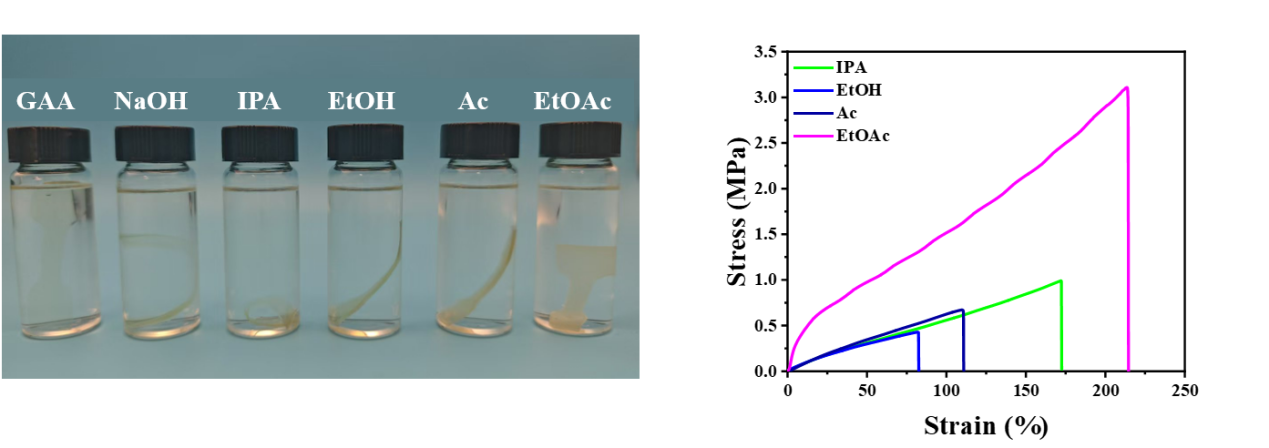


**Figure S13.** Solvent resistance of PHBA ionogels

Precise cutting yielded PHBA ionogel samples of uniform size, which were placed in 50ml sample bottles containing different solvents and stored at room temperature for five days before tensile performance testing. The severe water absorption of PHBA gel in GAA and NaOH solutions led to significant swelling, precluding accurate tensile data. Here, the PHBA ionogel demonstrated superior solvent resistance to EtOAC solvent, maintaining approximately 210% elongation at break and a tensile strength of 3.1MPa, with performance remaining essentially unchanged compared to the initial sample.

**
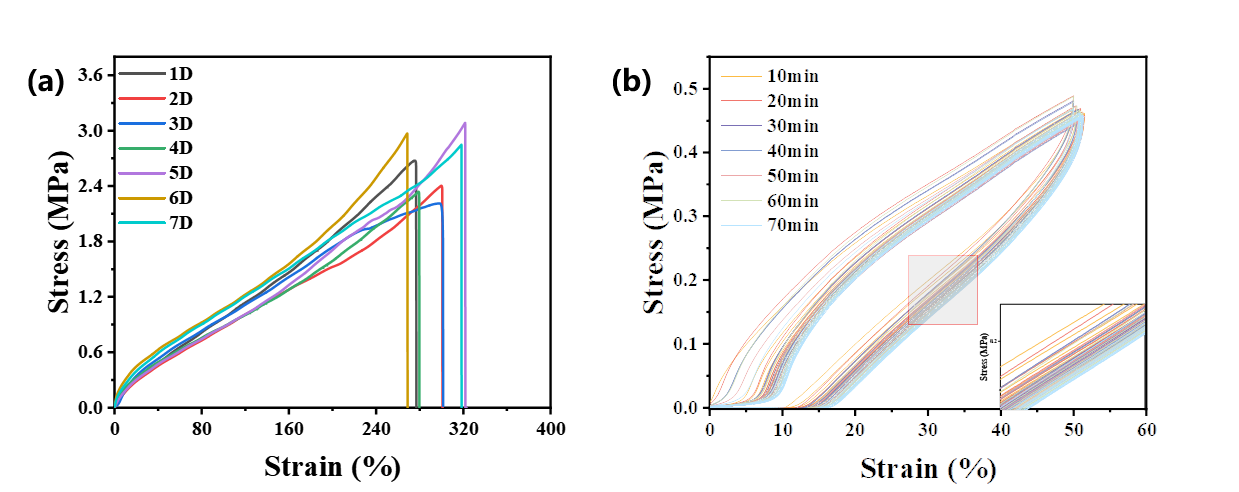
**

**Figure S14.** Mechanical stability of the PHBA ionogels.(a) Stress-strain curves after 7 days of storage at room temperature.(b) Tensile cyclic curves with 10 cycles at 50% strain, recorded at 10-minute intervals.

Uniform-sized PHBA ionogel samples were obtained through precise cutting and placed in petri dishes. The samples were stored at room temperature for 1 to 7 days. The material exhibited remarkable nonlinear mechanical responses with prolonged loading time, demonstrating dynamic stress evolution from 3D MPa to 5D MPa (where D denotes the base stress unit) accompanied by superelastic strain ranging 270%-320%. Particularly under quasi-static loading conditions, the maximum strain reached 3.2 times the original length, revealing exceptional deformation capacity.Furthermore, cycle tests were conducted using the mass structure instrument. The results showed that under room temperature conditions, the stress-strain curves of PHBA remained at a relatively high level over time, exhibiting only minor fluctuations.


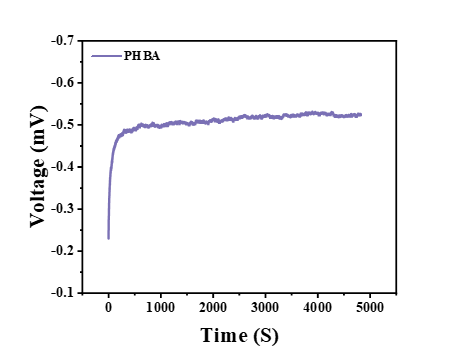


**Figure S15.** Thermal stability of the PHBA ionogels at room temperature

Because [AMIM]^+^ is anchored to the polymer chain, there is a large difference from the Cl-intermobility, PHBA ionogel can produce a certain voltage difference at room temperature / 60% RH, which can show extremely high stability.

**
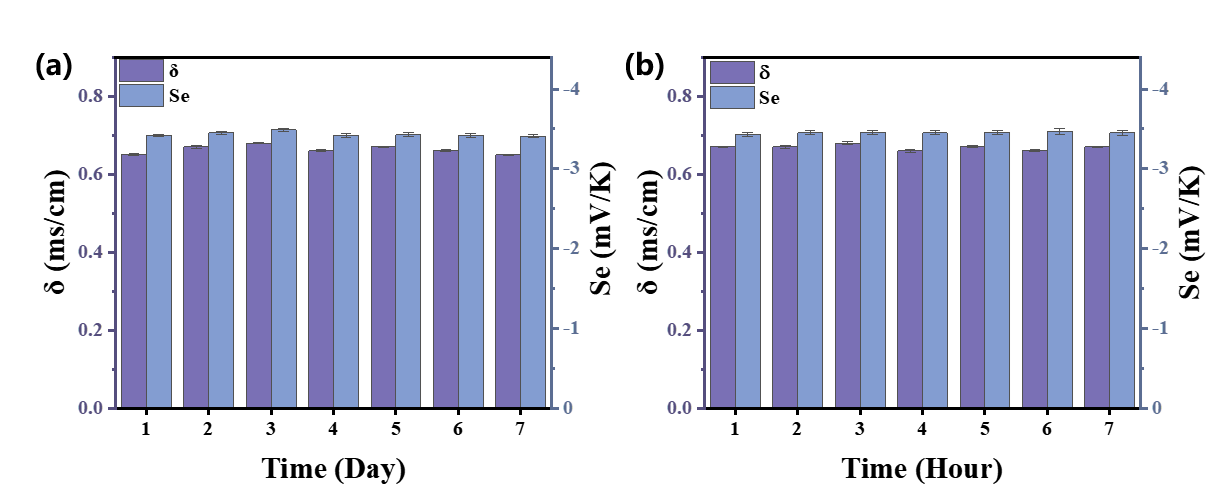
**

**Figure S16.** Stability of the Seebeck coefficient of the PHBA samples.（a）Stability of the Seebeck coefficient and conductivity over 7 days / 7 hours

Uniform-sized PHBA ionogel samples were obtained through precise cutting and placed in petri dishes to record mass changes over a continuous seven-day period. In the experiment, a temperature difference of 20 K was applied to one end of the samples (using a heating plate while maintaining the other end at room temperature, i.e., 10°C, 30% RH), and changes in the output voltage were recorded using a digital source meter. The results show that the conductivity of PHBA has only a small error and has high stability.


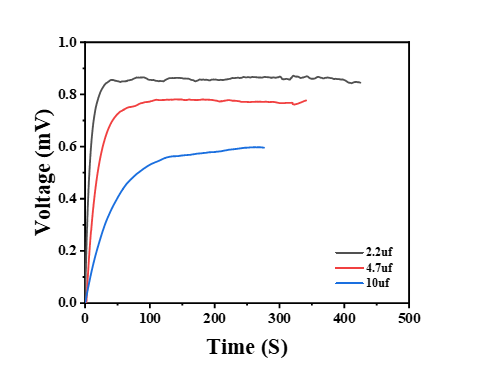


**Figure S17.** PHBA charges commercial capacitors after absorbing heat

After absorbing heat on the heating table, PHBA can be supplied in series with conventional capacitors, demonstrating the potential of PHBA in self-supply.


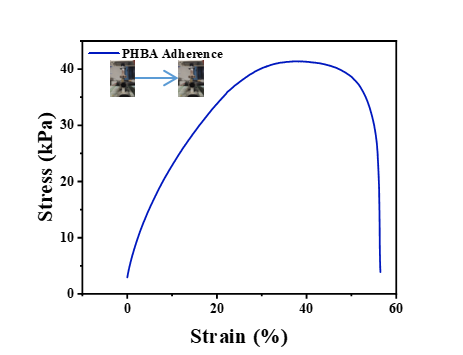


**Figure S18.** For PHBA adherence testing.

The PHBA material was precisely sectioned into 1 cm × 3 cm strips (denoted as Samples 1 and 2). Sample 2 was vertically stacked onto the terminal region of Sample 1 to establish an adhesive interface with a controlled contact area of 1 cm². After 5-minute interfacial consolidation under standardized contact pressure, the laminated specimen was mounted on a fixture for quasi-static axial tensile testing, revealing a peak tensile stress of 41.39 kPa.


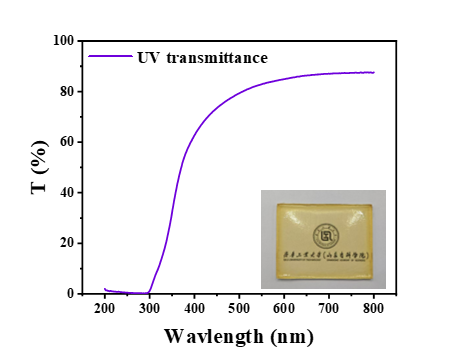


**Figure S19.** The 200 - 800 nm UV-light transmittance of the PHBA.

For the prepared PHBA sample, an ultraviolet spectrophotometer was employed to measure its ultraviolet light transmittance in the wavelength range of 200 - 800 nm. The results indicated that the sample demonstrated excellent ultraviolet resistance under the illumination of 200 - 300 nm. Moreover, it exhibited good light transmittance in natural light environment.


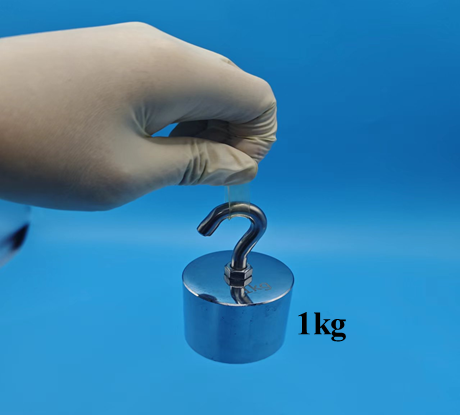


**Figure S20.** The PHBA load lifts a 1 kg weight.

A PHBA sample with dimensions of 1 cm × 5 cm was obtained by cutting with scissors. When the two ends of the sample are pinched, it can lift a 1 kg weight.

**References**

[1] J. Xu, H. Wang, X. Du, X. Cheng, Z. Du, H. Wang, *Chem. Eng. J.* **2021**, *426*, 130724.

[2] C. Mu, Y. Chen, Z. Dai, W. Li, J. Wang, X. Jiao, D. Chen, T. Wang, *Adv. Funct. Mater.* **2024**, *34* (17), 2313957.

[3] J. Wei, Y. Zheng, T. Chen, *Mater. Horiz.* **2021**, *8* (10), 2761-2770.

[4] J. E. Hyun, T. Lim, S. H. Kim, J. H. Lee, *Chem. Eng. J.* **2024**, *484*, 149464.

[5] H. Wang, Y. Mao, D. Ji, L. Wang, L. Wang, J. Chen, X. Chang, Y. Zhu, *Chem. Eng. J.* **2023**, *471*, 144674.

[6] K. G. Cho, S. An, D. H. Cho, J. H. Kim, J. Nam, M. Kim, K. H. Lee, *Adv. Funct. Mater.* **2021**, *31* (36), 2102386.

[7] X. Gou, J. Yang, P. Li, M. Su, Z. Zhou, C. Liao, C. Zhang, C. Dong, C. Li, *Nano Energy* **2024**, *120*, 109140.

[8] C. Zhou, X. Song, R. Wei, S. Liu, Z. Wu, H. Chen, *Chem. Eng. J.* **2024**, *499*, 155992.

[9] N. Yu, P. Liu, Y. Lin, A. Zhang, *Chem. Eng. J.* **2024**, *497*, 155046.

[10] L. Sun, H. Huang, L. Zhang, R. E. Neisiany, X. Ma, H. Tan, Z. J. A. S. You, *Adv. Sci.* **2024**, *11* (3), 2305697.

[11] Y. H. Pai, C. Xu, R. Zhu, X. Ding, S. Bai, Z. Liang, L. Chen, *Adv. Mater.* **2024**, 2414663.

[12] D. Lv, X. Li, X. Huang, C. Cao, L. Ai, X. Wang, S. K. Ravi, X. Yao, *Adv. Mater.* **2024**, *36* (17), 2309821.

[13] J. Sun, Y. Liu, J. Wei, P. Wei, T. J. C. E. J. Chen, *Chem. Eng. J.* **2024**, *485*, 149836.

[14] W. Zhao, Y. Zheng, M. Jiang, T. Sun, A. Huang, L. Wang, W. Jiang, Q. Zhang, *Sci. Adv.* **2023**, *9* (43), eadk2098.

[15] Z. Wang, H. Lv, Z. Gao, H. Song, *Chem. Eng. J.* **2024**, *498*, 155789.

[16] G. Fan, K. Liu, H. Su, Y. Luo, Y. Geng, L. Chen, B. Wang, Z. Mao, X. Sui, X. Feng, *Chem. Eng. J.* **2022**, *434*, 134702.

[17] S. Liu, Y. Yang, S. Chen, J. Zheng, D. G. Lee, D. Li, J. Yang, B. Huang, *Nano Energy* **2022**, *100*, 107542.

[18] M. Liao, H. Ma, N. Zhu, M. P. Jonsson, D. Zhao, *Adv. Sci.* **2024**, 2413093.

[19] Z. Liu, H. Cheng, Q. Le, R. Chen, J. Li, J. J. A. E. M. Ouyang, *Adv. Energy Mater.* **2022**, *12* (22), 2200858.

[20] W. Zhao, Z. Lei, P. Wu, *Adv. Sci.* **2023**, *10* (18), 2300253.
